# Global evolutionary history and spatio-temporal dynamics of dengue virus type 2

**DOI:** 10.1038/srep45505

**Published:** 2017-04-05

**Authors:** Kaifa Wei, Yuhan Li

**Affiliations:** 1 School of Biological Sciences and Biotechnology, Minnan Normal University, Zhangzhou 363000, China

## Abstract

DENV-2 spread throughout the tropical and subtropical regions globally, which is implicated in deadly outbreaks of DHF and DSS. Since dengue cases have grown dramatically in recent years, about half of the world’s population is now at risk. Our timescale analysis indicated that the most recent common ancestor existed about 100 years ago. The rate of nucleotide substitution was estimated to be 8.94 × 10^−4^ subs/site/year. Selection pressure analysis showed that two sites 160 and 403 were under positive selection, while E gene is mainly shaped by stronger purifying selection. BSP analysis showed that estimating effective population size from samples of sequences has undergone three obvious increases, additionally, Caribbean and Puerto Rico maintained higher levels of genetic diversity relative to other 6 representative geographical populations using GMRF method. The phylogeographic analysis indicated that two major transmission routes are from South America to Caribbean and East&SouthAsia to Puerto Rico. The trunk reconstruction confirmed that the viral evolution spanned 50 years occurred primarily in Southeast Asia and East&South Asia. In addition, phylogeographic association-trait analysis indicated that the viral phenotypes are highly correlated with phylogeny in Nicaragua and Puerto Rico (P < 0.05).

Dengue virus (DENV), a member of family *Flaviviridae*, is a positive-sense, single-stranded RNA virus. It has a genome size of approximately 11 kb, and the genome can be translated into a single, long polypeptide, which undergoes proteolytic processing into three structural proteins: capsid (C), membrane (M) and envelope (E); and seven nonstructural proteins: NS1, NS2A, NS2B, NS3, NS4A, NS4B and NS5. The viruses have been divided into four serotypes (DENV1–4). Although genetically closely related, the serotypes differ in antigenicity, with cross protection among them limited. The fifth variant DENV-5 has been isolated in October 2013, and yet it is only discovered in the sylvatic cycle^1^. Among the five serotypes, serotype 2 has been the most genetically diverse population^2^, and is the most frequent cause of dengue epidemic worldwide, which is known to be associated with severe dengue cases. Within a serotype, strain variation allowing viruses to be classified into genetically distinct groups based on genetic diversity and geographical distribution was called genotypes^3^. Outcome of disease may also depend upon the genotype involved. Some genotypes induce greater viremia and are more transmittable, hence having a higher potential to cause large epidemic. The viruses are causative agents for dengue fever (DF) and dengue hemorrhagic fever (DHF) and dengue shock syndrome (DSS) in humans. The greatest risk factor for development of DHF/DSS is secondary infection with a serotype different from that which caused the primary infection^4^. Antibody dependent enhancement (ADE) was considered as underlying threat during the second infection^5^, and sequential infections put people at greater risk for DHF and DSS. DHF occurs more frequently with Dengue virus type 2 (DENV-2) or DENV-3 infections in DENV-1 exposed individuals^6^. There is currently no specific therapy and clinical management is limited to supportive care. Development of dengue vaccine represents a major advance in the control of the disease and is considered a high public health priority. The first dengue vaccine, Dengvaxia (CYD-TDV) by Sanofi Pasteur, was registered in Mexico in December, 2015^7^.

The first record of probable dengue fever case can be traced back to Chin Dynasty (265–420 AD), which referred to a “water poison” associated with flying insect^8^. In the 1780s, the first clinically recognized dengue epidemics occurred almost simultaneously in Asia, Africa and the Caribbean, shortly after the identification and naming of the disease in 1779. From 1823 to 1916, DEN or DEN-like pandemics crisscrossed the globe, from Africa to India to Oceania to the Americas, which were regarded as a second series of the epidemics^9^. In 1944, DENV was first isolated from the sera of American soldiers in India. Since then, dengue epidemics have been mostly reported from Southeast Asia, particularly after the end of World War II^10^. Severe dengue (DHF) was first recognized in the 1950s in Philippines and Thailand. It was not until 1981 that DENV-2 strain of Asian origin was introduced into Cuba with a significantly increased severity during subsequent epidemics, causing the first epidemic of DHF in the Americas^11^. The disease is now endemic in more than 100 countries in the WHO regions, among which the Americas, South-East Asia and Western Pacific are the most seriously affected. In 2008, cases across these three regions exceeded 1.2 million. In 2010, Puerto Rico experienced the largest outbreak in its history with 5382 confirmed infections and 20 deaths. Nearly 2.4 million cases were reported annually in the member states of 3 WHO regions (http://www.who.int/en/) in 2010, 2013 and 2015. The number of new cases arising each year is still increasing globally.

Dengue is transmitted by female mosquitoes mainly of the species *Aedes aegypti*, to a lesser extent, *Ae. albopictus*. The mosquitoes also transmit Zika, Yellow fever, and Chikungunya infection. The disease has been widespread throughout the tropics and now a global threat. Nearly all tropical/subtropical nations had reported explosive increases. Geographical limits of dengue fever transmission are strongly determined by climate such as rainfall and temperature^12^. In addition, air travel has been considered as a crucial factor driving the intracontinental spread^13^. One recent estimate indicates 390 million dengue infections per year (95% credible interval 284–528 million), of which 96 million (67–136 million) manifest clinically^14^. Today, severe dengue disease burden affects most Asian and Latin American countries and DHF/DSS has become a leading cause of death among children in these regions^15^. Due to the fact that dengue viruses have evolved rapidly and spread worldwide, genotypes associated with increased virulence have expanded from South and Southeast Asia to the Pacific and Americas^16^.

In recent decades, reports of dengue infections in human migration from the tropics and subtropics have been increasing. Not only has the number of cases increased but also the explosive outbreak spread to new regions^17^. Many phylogenetic studies on DENV-2 have documented the viral spread within individual countries or specific regions^18^,^19^, so the geographic origins, migration patterns have been poorly investigated. Additionally, E protein is the major protein on the surface of the virion, a N-glycosylated dimeric membrane fusion protein that mediates virus binding and fusion to host cell membrane^20^,^21^, and thus might be under strong selective pressure from the host immune response. Most importantly, E protein contains the main epitopes for vaccine development and is used as a reagent for diagnostic purposes. Therefore, we focus on evolutionary analysis of E protein and try to reveal the population dynamics, migration direction and geographic origin of DENV-2 in global.

## Results

### Phylogenetic analyses

**Figure 1:**
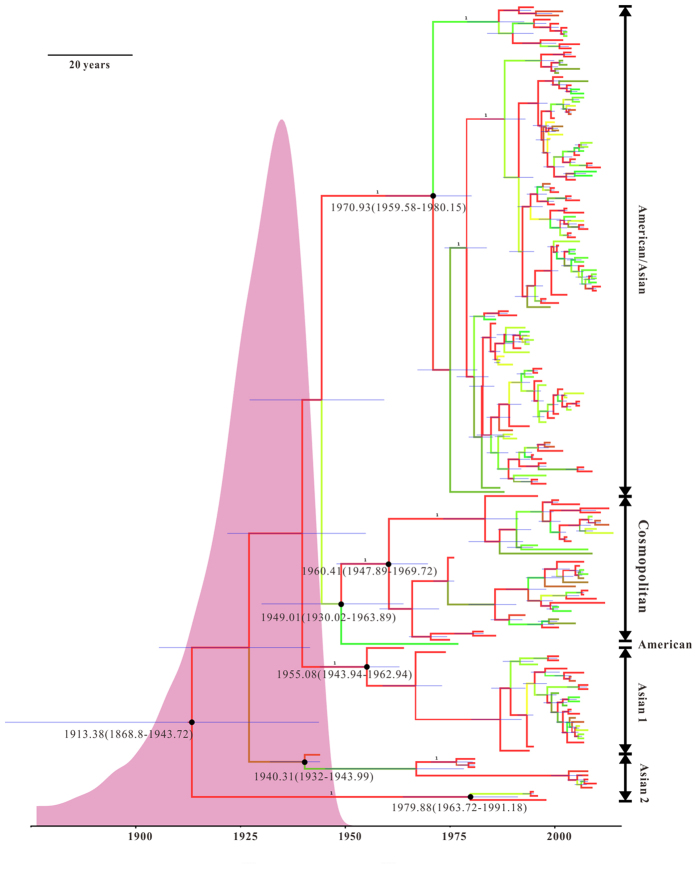
Maximum clade credibility tree from Bayesian analysis of Dengue type 2 viruses. Maximum clade credibility (MCC) tree was constructed using BEAST program. Each branch is colour coded to indicate the posterior probability (PP) value, Blue bars at nodes indicate 95% highest probability density (HPD). The mean time to the most recent common ancestor (TMRCA) is shown in each principal node (mean and 95% HPD). Genotypes are indicated on the right. The part of pink shows the posterior probability densities for the estimated age of the most recent common ancestor. An identical MCC tree with strain names is shown in Fig. S3.

To determine the phylogenetic relationships among dengue-2 viruses, phylogenetic trees based on E gene sequences were inferred using the maximum likelihood (ML) and the Markov chain Monte Carlo (MCMC) methods. The ML tree allows clearly distinguish the topological structure, and the bootstrap values (>75) are displayed on the tree with rainbow color (Fig. S1). The isolates derived from the same geographic area tend to cluster together, which makes the strains displayed a marked geographic distribution. Generally, the isolates in the tree can be divided into five genotypes: American/Asian (AM/AS), Asian type 1 (Asian 1), Asian type 2 (Asian 2), Cosmopolitan, and American (Fig. S1). Further observation indicated that geographic-close regions share the same DENV-2 genotype and this would serve as a control to show that DENV-2 genotype variation is a major underlying determinant of epidemics. In genotype AM/AS, the strains may be separated into two distinct subgroups, which are mainly from the countries in the Americas and some from Southeast Asia (10 isolates from Viet Nam and 2 isolates from Cambodia). While the majority of Southeast Asia strains were present in Asian 1. Asian 2 is composed of strains from New Guinea C (NGC), Taiwan and other regions. The Cosmopolitan genotype has a wide geographical distribution, including Southeast Asia, India, China, Guam, Saudi Arabia and Burkina Faso. The American genotype has one strain (DENV-2/ID/1183DN/1977). In order to verify the reliability of grouping, we have included three American genotype viral sequences which have been previously reported, such as DENV-2/Tonga/1974 (AY744147), DENV-2/CO/BID-V3358/1986 (GQ868592) and DENV-2/PE/IQT2913/1996 (AF100468), as the reference for the genotype (Fig. S2). Hence, it is convincing that we named the American genotype solely based on one virus isolated from Indonesia. Maximum clade credibility tree depicted the proportional relationship between branch length and time, representing the genealogy of the virus strains by analyzing 194 sequences. Comparatively, the topology of the MCC tree is congruent with that of the ML tree (Figs 1, S1 and S3). Interestingly, when compared to the tree topology of influenza virus characterized by a ladder-like trunk and short terminal branches, we obtained a unique equidistant phylogeny with short terminal branches. The equidistant topology indicated that all prevalent lineages in the study are transient under a selection enhancing host immunity.

### Natural selection

**Figure 2:**
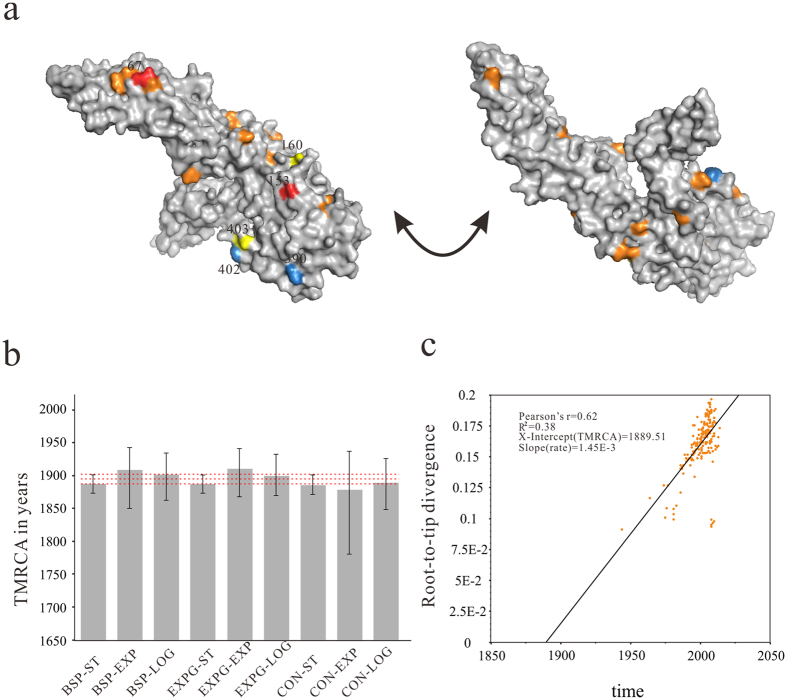
Mapping of positively selected and glycosylation sites on the E protein and TMRCA comparison and Root-to-tip regression of ML tree. (**a)** Three-dimensional structure of E protein. Red indicates N-linked glycosylation sites; Orange indicates the O-linked glycosylation sites; Yellow indicates the positively selected amino-acid sites identified; Blue indicates the positive selected sites which is the same as previously reported. (**b**) Robustness of key parameter estimates to the choice of coalescent and molecular clock priors in Bayesian inference framework. Vertical boxes show mean parameter estimates and vertical bars represent the corresponding 95% confidence interval (CI). The red dashed lines represent the mean and 95% CIs of the parameter estimates. Horizontal axis shows the Tree Prior-Clock model. BSP, Bayesian skyline plot; EXPG, Exponential Growth; CON, Constant Size; ST, Strict Clock; EXP, Exponential relaxed clock; LOG, Lognormal relaxed clock. (**c**) Linear regression analysis of the root-to-tip regression of genetic distance against sampling dates for the E gene sequences.

To avoid high rates of false positives, the recombinant sequences were removed before quantifying selection pressure. Putative positively selected sites were identified using Single-Likelihood Ancestor Counting (SLAC), Fixed Effects Likelihood (FEL), Random-Effects Likelihood (REL), Mixed Effects Model of Evolution (MEME) and Fast Unconstrained Bayesian AppRoximation (FUBAR). Site found to be statistically significant for selection pressure by two methods was considered positive (P < 0.1, posterior probability (PP) > 0.9, or Bayes Factor >20). Four codons under positive selection were identified by REL method, whereas MEME analysis detected ten diversifying selection sites. Taken together, positions 160 and 403 were identified as being under potential diversifying selection (Fig. 2a). Unfortunately, the SLAC and FEL analyses did not find any sites affected by positive selection but suggested 191 sites and 255 sites influenced by negative selection, respectively. Also, the FUBAR method did not detect any codons under pervasive diversifying selection exceeding a PP > 0.9. It should be noted that no positive selection was detected in sites 390 and 402 in our study, which is located within or adjacent to the putative receptor-binding domain (domain III) implicated as a potential virulence determinant in mice in American DENV-2 genotype^22^,^23^. Obviously, most of sites in the E gene are under strong negative selection, indicating strongly conserved. The purifying selection shows a typical highly adapted phenotype, which is probably caused by constraints imposed by protein structure and function. Then, we correlated the positively sites with the time-scale of DENV-2 migration, most codon positions were under negatively selection. Comparatively, selection pressure in the early viruses involved an increase in amino-acid mutations, leading to the increased positive selection most likely in response to host immune pressure. While the decreased positive selection or increased negative selection were observed in the most recent period (Table S1, Fig. S4). Totally, the positive selection on DENV-2 is relatively weak when compared to the viruses that appear to be under strong host immune pressure, such as influenza A virus^24^,^25^ and hepatitis C virus^26^. Glycosylation focus on the importance of viral virulence and immune evasion, so we predicted glycosylation sites in envelope glycoprotein. Probable 23 O-linked and 2 N-linked sites were presented in Table S2 and Fig. 2a. The N-linked glycosylation site 153 is conserved in most flaviviruses, while the position 67 is unique for dengue viruses. All glycosylation sites detected in this study are not under positive selection. Collectively, purifying selection may be more important in shaping E protein evolution, leading functionally and structurally conserved.

### Evolutionary rate and divergence date estimate

After molecular clock and demographic models selection using Akaike information criterion through MCMC (AICM), the relaxed molecular clock (uncorrelated exponential) and an exponential growth tree prior are the best combination in our data analysis (Table 1, Fig. 2b). For the sampled population, the mean evolutionary rate was estimated to be 8.94 × 10^−4^ substitutions/site/year (subs/site/year) (95% highest posterior density (HPD) interval = 7.39 × 10^−4^ to 1.04 × 10^−3^), and the estimated mean time to most recent common ancestor (TMRCA) of the tree root was 100.62 years (95% HPD: 70.29, 145.20 years), indicating that the pathogen existed since ∼1913. In the previous section, we reported that the five genotypes are circulating in the world. Estimation of divergence dates for the genotypes showed that AM/AS (n = 119) was introduced in 1970.93 (95% BCI: 1959.58, 1980.15), Cosmopolitan genotype (n = 36) in 1960.41 (95% BCI: 1947.89, 1969.72), Asian 1 (n = 26) in 1955.08 (95% BCI: 1943.76, 1962.94), Asian 2 (n = 12) in 1924.03 (95% BCI: 1892.23, 1943.74), and genotype American (n = 1) in 1977. While in the root-to-tip regression analysis for DENV-2 population, the nucleotide substitution rate was estimated to be 1.45 × 10^−3^ nucleotide subs/site/year, and the TMRCA could date back to 1889.51 (Table 1, Fig. 2c). The analysis indicated that DENV-2 evolved almost 1.6-fold faster and diverged approximately 24 years earlier than those estimated by using the BEAST program. Although there are some slight differences between two methods, they still fall within the confidence interval.

### Reconstruction demographic history

**Figure 3:**
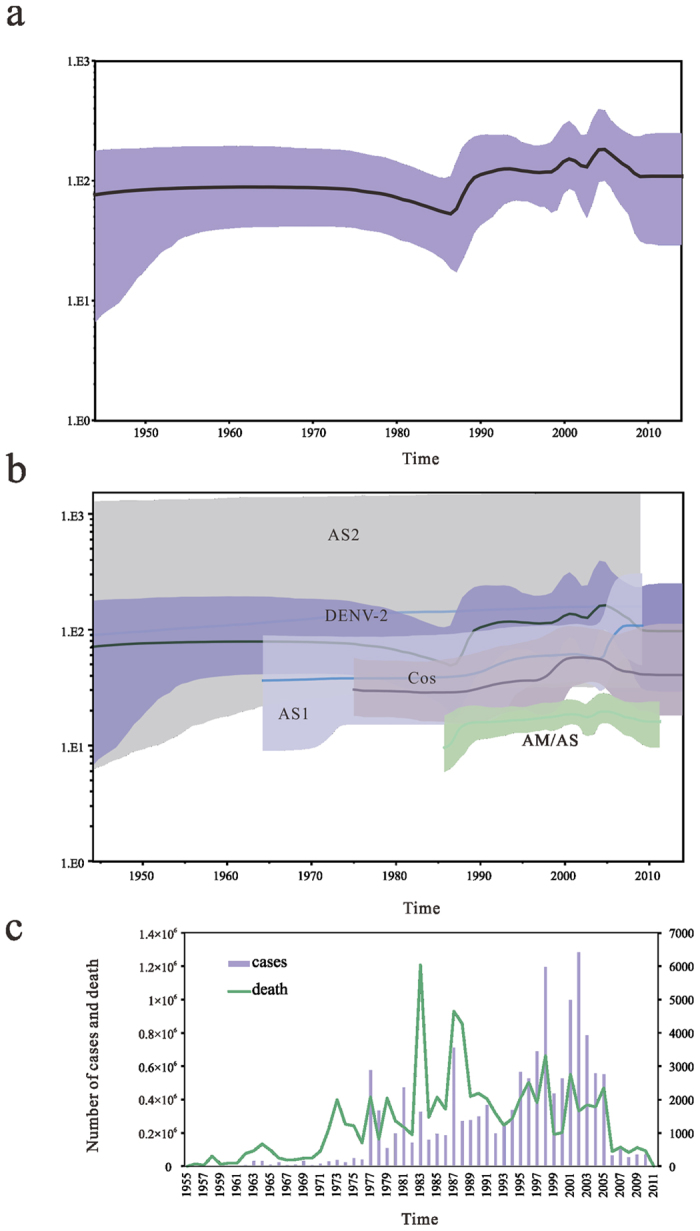
Evolutionary dynamics of dengue virus type 2. (**a**) Bayesian skyline plot (BSP) shows the changes in effective population size of DENV-2 over time. The thick solid line indicates the median estimates, and the purple area displays the 95% HPD. (**b**) Comparison of Bayesian skyline plot between DENV-2 and each genotype. The median estimates are represented by the solid lines and 95% high posterior densities are shown in the color regions. AS1, Asian 1; AS2, Asian 2; AM/AS, American/Asian; Cos, Cosmopolitan. (**c**) The number of confirmed dengue cases (purple bars), and the number of human death (green lines) of DENV in global during 1955–2011.

To explore the demographic history of the sampled population and genotypes over the study period, the relative effective population size over time was inferred by analyzing the genetic diversity using the Bayesian skyline model. As shown in Fig. 3a, the population has had a steady size until a first decay inferred around 1975. The decrease was followed by a sharp increase started around 1987–1990, coinciding with the period when genotype AM/AS viruses were introduced (Fig. 3b). This genetic diversity reached a plateau that remained steady until the end of 1997. The geographic expansion of the DENV-2 entailed two major waves since 1997, and the first reached its peak before 2001. The effective population size of three genotypes remaining relatively stable during the period, the Cosmopolitan genotype may be the major contributor to the genetic diversity of DENV-2 (Fig. 3b). Correspondingly, Philippines experienced its worst dengue outbreak during 2000–2001^27^. The second sharp increase in diversity occurred reaching higher levels of genetic variability than that of the first, peaking around 2005, and approximately 14,000 people were infected during the worst dengue outbreak on record for Singapore in the year^28^. The diversity of DENV-2 declined sharply during 2005–2009, which may be the use of mosquito repellent to protect human against mosquito and to break the chain of transmission^29^, but the genetic diversity in Asian 1 showed an upward trend in this period. The population size of DENV-2 remained stable after that. Our observation showed that DENV genotype variation is a major underlying determinant of epidemics. To further understand the changing ecology and epidemiology of the disease, the epidemiological data of DENV serotype from the global were collected from WHO (Fig. 3c). Although the event of exponential increase in global dengue cases during 1976–1977 was not detected by the Bayesian skyline plot (BSP) analysis, the analysis is generally consistent with the epidemiological dengue cases of all four DENV serotype.

**Figure 4:**
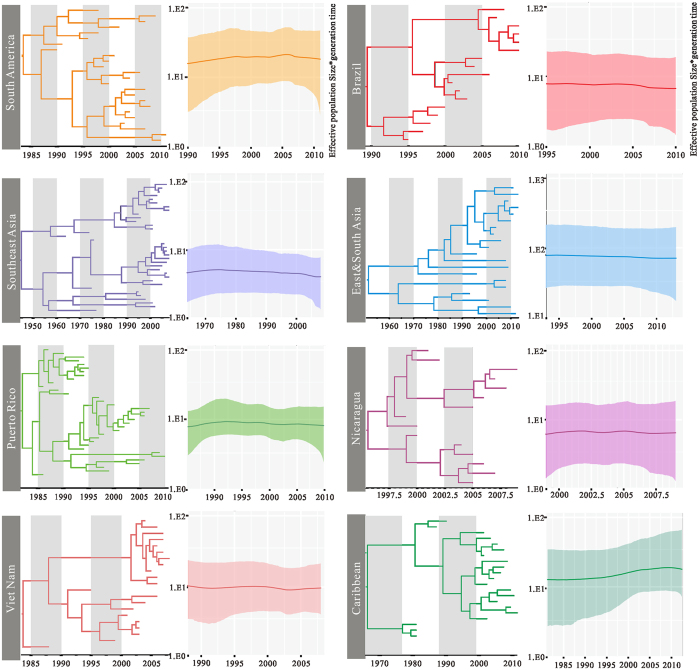
Comparative phylogenetic analyses and population dynamics of Dengue virus type 2 circulating in different geographical regions, 1964–2013. Phylogenies were inferred using the uncorrelated exponential relaxed clock model, and relative genetic diversity was estimated using the Gaussian Markov Random Field (GMRF) model. The x-axis is in units of year, and the y-axis represents the logarithmic scale of Neτ (where Ne is the effective population size and τ is the generation time).

To infer the time-resolved phylogeny in representative areas, an estimation of the relative change in the population size of DENV-2 over time was made from Gaussian Markov random field (GMRF) Bayesian Skyride coalescent model, which differs from the BSP by not requiring the specification of a user-defined prior on the number of population size changes in the history of the sample. We compared the relative genetic diversity of 8 different geographic regions with coinciding epidemic trends, representing the tropics and subtropics areas (Fig. 4). The effective population sizes of the regions are keep stable growth except for those in Puerto Rico, the Caribbean and South America. Puerto Rico shows a pattern of small fluctuation, and the wave began in early 1986 (median = 8.68). Relative genetic diversity of Caribbean increased rapidly from 1981 to 2003 (95% CI: 4.26–38.74), then remained almost constant in the period 2004 to 2011 (95% CI: 8.03–63.84). And South America region typically shows higher levels of relative genetic diversity (median = 16.95).

### Phylogeographic and migration pattern analyses of DENV-2

**Figure 5:**
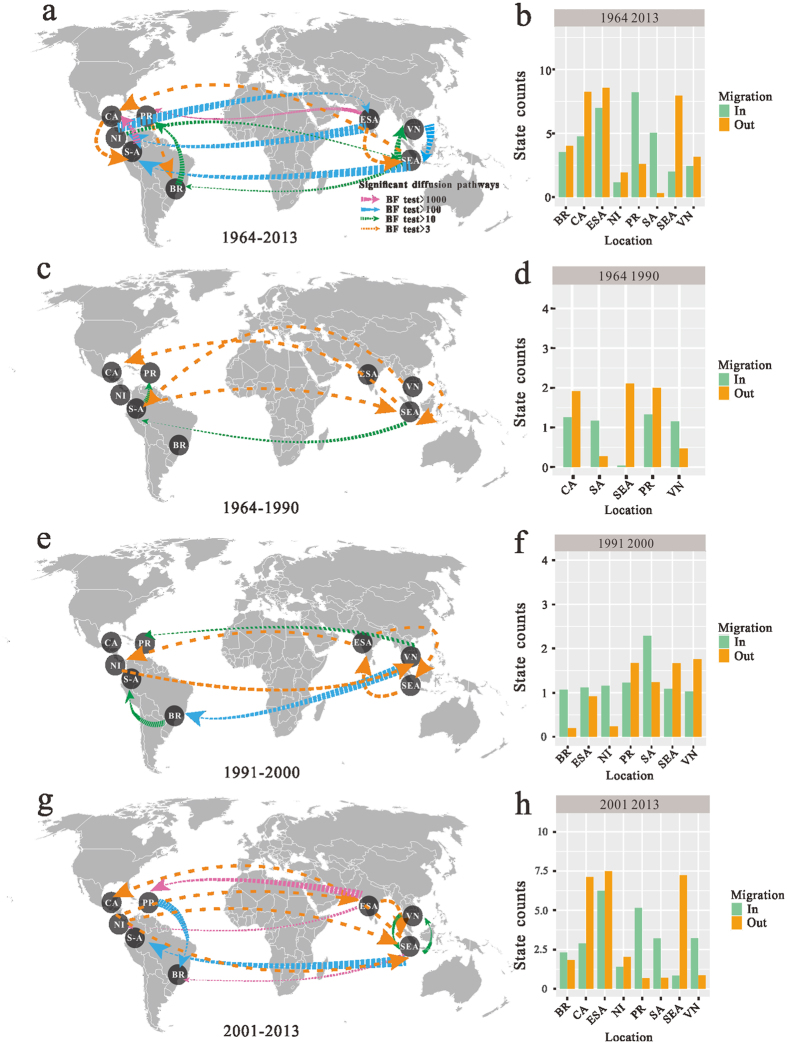
Spatial-temporal diffusion of Dengue type 2 virus. Distributions of temporal-spatial diffusion pathways and histograms of total number of state transitions using “Markov Jump” model for (**a,b**) 1964–2013, (**c,d**) 1964–1990, (**e,f**) 1991–2000, (**g,h**) 2001–2013. Significant pathways from one location to another are indicated on the maps. Pink arrow represents very strongly supported rate with BF ≥ 1,000; Blue arrow represents strongly supported rate with 100 ≤ BF < 1,000; Dark green arrow, supported rate with 3 ≤ BF < 100; Orange arrow represents the support with BF > 3. BR, Brazil; ESA, East&South Asia; NI, Nicaragua; CA, Caribbean; SA, South America; SEA, Southeast Asia; PR, Puerto Rico; VN, Viet Nam. The map source is downloaded freely from FREE WORLD MAPS (http://www.freeworldmaps.net/pdf/world/miller-world.pdf).

As phylogeographic analysis of DENV transmission regionally is conceivable, we first carry out a phylogeographic analysis within Asia. Locations that are epidemiologically linked are identified using the Bayes Factor (BF) test under Bayesian Stochastic Search Variable Selection (BSSVS). The transition between Singapore and China received the strong support by the BF criterion (BF = 708.7) (Fig. S6a). In order to understand the migration process acting along DENV-2 phylogeny in Asia, Fig. S6b projects each of the branches of the MCC phylogeny onto a geographic map. The earliest routes began in India, continued to China and Philippines and then Cambodia before 1970. In 1971–1985, the virus appeared to spread from China to Viet Nam and from India to Indonesia. In the next few years, it is important to note that the circulation of the virus resulted in a very high incidence of epidemics in the three countries (Thailand, Viet Nam and Cambodia) during 1986–2005. After 2006, the virus had expanded their geography distribution to Pakistan. Subsequently, we expanded to transcontinental analysis to understand the geographic origins of the DENV-2 virus in the world. The phylogeographical analysis shows that Nicaragua, Puerto Rico, Brazil, Caribbean, South America, Southeast Asia, Viet Nam and East&South Asia played central roles in the dispersal of DENV-2. To illustrate the spatial diffusion of the virus within the tropics and subtropics, a joint analysis of discrete trait models was performed to estimate the overall genetic transmission process. Figure 5 shows the maps of the routes when calculating a BF for the most significant non-zero rates, and we used a BF cutoff of 3 to determine significance. And the rates of viral migration across the eight regions were estimated. Fourteen migration events with strongly support were summarized in Fig. 5a and Table S3. Among them, two significant migration links were established with mean rates from 0.92 to 0.937 (decisive support with BF > 1,000), with one originating from South America to Caribbean, and another from East&SouthAsia to Puerto Rico (Fig. 5a, Table S3), state counts support the idea that East&SouthAsia may have acted as a seeding population (Fig. 5b).

For a more detailed understanding the routes of invasion of DENV-2, eight different data sets were used to represent the global epidemic periods: 1964–1990, 1991–2000, 2001–2013. During 1964–1990, there are six significant migration links with higher supported diffusion rates (Fig. 5c), and state change counts clearly reflected this dynamic with outward migration from Southeast Asia and inward migration from Puerto Rico dominating (Fig. 5d). From 1991 to 2000, one strongly supported pathway (BF > 100) from Viet Nam was apparent, indicating migration from Viet Nam to Brazil (Fig. 5e), meanwhile state counts suggest that a migration out of Viet Nam is probable (Fig. 5f). In the period from 2001 to 2013, three strong epidemiological links (BF > 1,000) from East&South Asia to Puerto Rico and Nicaragua and from Southeast Asia to Brazil were illustrated in Fig. 5g, and these were supported by the state change counts with migration from East&South Asia being much greater than any other location included in our analysis (Fig. 5h). The inferred spatial dynamics suggested that multiple geographical regions might act as a potential seed for local epidemics. Additionally, we reconstructed genealogical tree with time-scale and inferred ancestral locations of each branch through a Bayesian phylogeography framework. The most probable location of each branch of the tree was assigned to different colors and the calibrating time-scale was shown on the bottom, and the Southeast Asia has the highest posterior probability of being the location of the tree root (Fig. S5), indicating that the five genotypes seem to have originated from the Southeast Asia.

**Figure 6:**
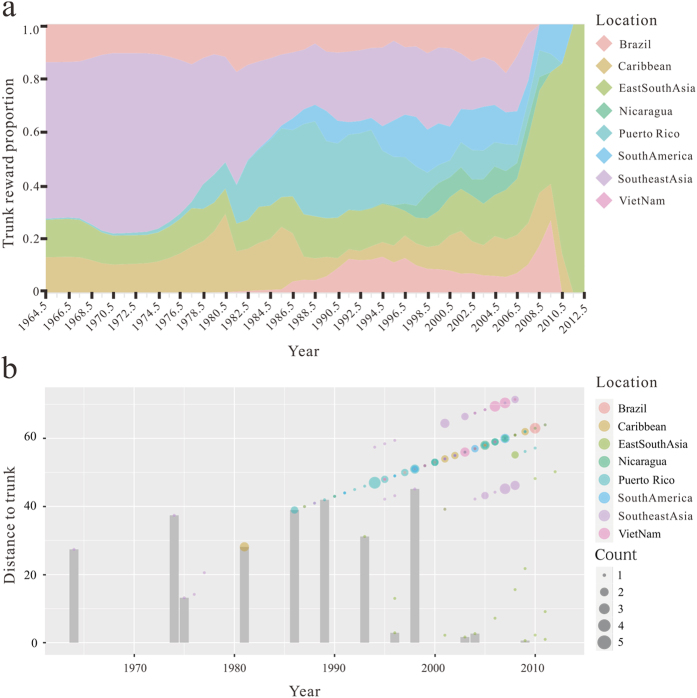
The geographic location of the trunk estimated for each location over time. (**a**) The trunk reward proportion for each geographic location from 1964 to 2013. Shaded areas represent the trunk proportions over time for the eight geographical locations. (**b**) Distance to the trunk measured in terms of years. Scatter plot represents the distance to trunk of the tree. With the sample size increasing, the dot size becomes larger. The height of each point on the y-axis shows the mean distance to the trunk and the bars identify the closest strain to the trunk every three years. Bars are colored according to eight sampled regions.

A structured coalescent approach was used to calculate the proportion of the trunk for each geographic region. A higher proportion implies that the corresponding region is more likely to be the source of the virus. The mean proportion and credible interval of the trunk assigned to each geographic region were summarized in Table 2. The trunk of the genealogy predominantly resides in Southeast Asia (32%) and East&South Asia (15%) from 1964 to 2013 (Fig. 6a). As previously established, migration patterns near the root of the tree support Southeast Asia as source populations (Fig. S5). We observed that samples from the Southeast Asia during the period from 1964–1985 coalesce to the trunk of the genealogy rapidly, and from 1986 to 1993, Puerto Rico samples are closer to the trunk than samples from others (Fig. 6b).

To assess the strength of geographical association with sampling locations across the entire tree, the global trait association tests of phylogeographic structure were performed and the null hypothesis of no association between sampling location and phylogeny is nearly always rejected. The analysis shows a very strong geographic clustering of strains by area of origin (P = 0 for both association index (AI) and parsimony score (PS) statistics). The monophyletic clade (MC) statistic was used to test the extent of phylogenetic clustering of individual regions, the result showed that population subdivision was significant for most of the localities (P < 0.05). The most significant geographic correlation of lineages was found in Nicaragua and Puerto Rico, reflecting predominantly local evolution in these regions (Table 3).

## Discussion

The dengue virus has become a leading cause of illness and death in the tropics and subtropics, emerging as a serious public health problem in the world. Some 50–100 million new infections are estimated to occur annually in more than 100 endemic countries, with a documented further spread to previously unaffected areas (http://www.who.int/). The viruses co-circulate in many countries with multiple serotypes, because infection with one serotype does not provide long-term protection against the other serotypes. For a long time, there is no specific antiviral therapy or vaccine in clinical use for the disease. But now, the first dengue vaccine approved in Mexico on December 9, 2015^7^. In spite of this, continuous surveillance of the virus can facilitate the early detection of novel emerging variants, and acts as a fundamental part of integrated preventive strategy. Previous studies were mainly about specific country and region^7^,^30^,^31^,^32^,^33^, or only focused on certain genotype^19^. Therefore, the challenge is to analyze evolutionary history and geographic spread of the virus globally. We utilized a larger and more temporally and geographically spread data to understand epidemics and endemic diseases, and provided effective control for epidemiology.

In this study, a consensus ML tree and Bayesian tree were constructed using 194 nucleotide sequences isolated from geographically distinct regions. However, low bootstrap support values of >60% and posterior probability of >0.53 are shown above the branch with one strain (DENV-2/ID/1183DN/1977) (Fig. 1, Fig. S1). As we have mentioned previously, we have discussed that the strain from American genotype with the reported strains (DENV-2/Tonga/1974, DENV-2/CO/BID-V3358/1986 and DENV-2/PE/IQT2913/1996) as the reference (Fig. S2). The result indicated that the isolate from Indonesia can be named the American genotype. The strains from America share a common ancestor with the genotypes circulated in Cambodia and Viet Nam, that is, AM and AS lineages have co-circulated at certain point in the past^34^. The Vietnamese sequences sampled between 1988 to 2006 (n = 10) belong to the AM/AS genotype, while the sequences collected during the period from 2004 to 2008 are included in Asian 1 genotype. Our sampling illuminated the replacement of the previously dominant AM/AS genotype by Asian 1 viruses in 2007, while it was once reported that the lineages were replaced by Asian 1 during 2003–2007^35^. Vietnamese sequences sampled between 2003 to 2006 belong to the AM/AS genotype in our analysis, which may be due to the difference in sampling time. The phylogeny indicates that Asian 2 has different sources, including three strains from Cuban, one from Papua New Guinea, two from Taiwan and so on. This confirmed the results of the previous study that the strain causing the 1981 Cuban epidemic had great similarity to the prototype NGC strain isolated in 1944^36^,^37^. The Indian strains isolated per-1971 belong to the American genotype^2^, while the strains isolated during and after 1996 belong to the Cosmopolitan genotype, indicating that genetic variation occurred among populations circulated in India. Further, the coalescent analysis based on the sampled sequences (excluding sylvatic strains) revealed that our TMRCA estimate is 238 years younger than that proposed by Walimbe AM^18^, that is to say, our estimate for all sequences approximately dated back to 1913. And the mean of substitution rate was estimated to be 8.94 × 10^−4^ subs/site/year, higher than that calculated in previous study^38^. What causes differences between the estimates? A lot of sylvatic strains and more endemic strains were included in the previous analyses, resulting in an earlier TMRCA value and lower the evolutionary rate.

E protein mediating receptor binding and endosomal fusion is the major antigenic determinant of DENV. Some amino acid sites may be located in or near B- or T-cell epitopes for neutralizing antibody and T-cell responses, and the positive selection acting on the sites is associated with immune evasion. In some of the earlier studies, positions 360, 390 and 402 were reported to be under positive selection^9^,^39^,^40^. Although the mutation N → D390 exists in American genotype, no statistically significant positive selection was detected at the site by five methods. The site 390 has been identified as a hot spot for DENV-2, because it may be crucial in determining the virulence. The site 402 locates within the stem-anchor region adjacent to the putative receptor-binding domain, which confers neurovirulence in mice^23^. Our analysis showed that two previously unreported sites (160 and 403) were under weak positive selection detected by two methods simultaneously. The positively selected site 403 may have a similar function with site 402, but the validation need more experimental evidences. Although previous studies reported sporadic positive selection in E protein amongst four DENV serotypes^21^, purifying selection is clearly the dominant evolutionary pressure acting on DENV. DENV-2 E protein evolved to a high level of fitness for alternate infection between mosquitoes and primates, resulting in most amino acid changes being deleterious and purified quickly from the population by selection^9^. As a consequence, we believe that there is a limit to TMRCA estimation for rapidly evolving viruses and strong purifying selection can lead to an underestimation of viral origins.

Phylodynamics of dengue virus focuses on transmission dynamics in an effort to shed light on how the dynamics impact viral genetic variation, and understanding the population dynamics of the virus can provide important insights into epidemiology and virus evolution. Our Bayesian skyline plots showed the demographic history of DENV-2, a decay was observed during 2005–2009. While population size of Indian Cosmopolitan strains remained almost constant in the period 2005 to 2009^41^, the changes in genetic diversity of other genotypes may resulted in the decay of DENV-2 population size. Furthermore, the event of exponential increase in global dengue cases during 1976–1977 was not detected by the DENV-2 BSP analysis, it is possible that the increase of cases is due to other three DENV serotypes. Because epidemiologic data for single serotype is unavailable in the public database, we are unable to obtain the data for DENV-2 serotype. Nevertheless, the overall temporal trend of the epidemiological dengue cases of all four DENV serotypes is generally consistent with the DENV-2 BSP analysis, indicating that DENV-2 serotype remains predominant in the four serotypes. Epidemiology is a central feature of public health policy and disease management, and prompt case detection and appropriate clinical management can reduce the mortality from severe dengue. In fact, dengue disease has been neglected by the scientific and pharmaceutical industries with no new drug development^42^. Management of dengue disease relies on early diagnosis and prevention with vaccines, but the cause of concern for the local medical community is often unaware of diagnosis and management of the new disease, which leads to avoidable morbidity and mortality.

While Mir *et al*.^33^ investigated DENV-2 migration using similar tools, the study was geographically limited to the Americas including the Caribbean. By contrast, our study attempted a global phylogeographic analysis of DENV-2 transmission. Yet phylogeographic analysis of DENV-2 transmission regionally is conceivable, it is difficult to conceive how transmission can occur via long distances over short periods. Even if globalization and travels were taken into account, one would expect to see a concentration of virus dissemination within neighbouring regions, in particular the ones with a smaller time scale. Therefore, we conducted additional analysis within Asia for emergence and dissemination of DENV-2. Subsequently we inferred the evolutionary history in the context of global spread on a much larger scale than the previous study for transcontinental analysis. The aim of our phylogeographic research was to reconstruct the spatial and temporal dynamics based on the analysis for the data set composed of 188 sequences sampled from 1964 to 2013. We have shown that the genetic population structure of DENV-2 arises in part from global migration dynamics, with the most important contributions from South America and Southeast Asia, and the Southeast Asia might be the geographical origin of DENV-2, which is supported by Rico-Hesse *et al*.^43^. The migration route from South America to Caribbean and East&South Asia to Puerto Rico were strongly supported by BF test. This will lead to geographical expansion of the disease, just like the epidemics expanded from Southeast Asia countries to India^44^. Our results in turn could guide the policy makers for controlling the global dengue transmission, highlighting the usefulness of phylogeography for public health surveillance and the analysis of disease transmission within the regions. Besides anthropogenic factors significantly influencing the DENV migration, some factors involved in the viral dispersal, such as unplanned rapid urbanization, proliferation of mosquitoes, bad sewer and waste management, increased air travel and global warming.

Our use of the structured coalescent model to analyze DENV evolution represents a significant step forward over previous methods. More importantly, the sampling date, sampling location and an underlying model of the demographic process were incorporated into coalescent method, the details will allow for more accurate reconstruction. By analyzing a large number of sampled trees, our estimates of the trunk location over time have a degree of confidence associated with them, confidence intervals are attached to proportions in all regions. Additionally, it is considerable that samples from Southeast Asia are the closest to the trunk, because of this, we can assume that the trunk of the genealogy resides in Southeast Asia. During 1986 to 1993, Puerto Rico samples are closer to the trunk than the strains from other regions, but are not close to the trunk in absolute terms.

Mathematical models have been recognized as useful epidemiological tools in exploring complicated relationships underlying infectious disease transmission processes. The limitation is the fact that modelling exercise is inevitably based on assumptions. One can argue about whether they are too simplistic or impractical and therefore hamper quantitative conclusions about the reality. The reality is always more complex, so epidemiology or DENV transmission will always involve some approximation, and parameter estimates will always involve a certain degree of error and bias. As long as the scientist understands the nature of the problems, approximation, error, and bias do not, by themselves, invalidate the utility of an estimate. The major limitation about the study is that reconstruction of the viral movement pattern was limited to transmission among population sampled, while unsampled populations may play a role in the spread and persistence of DENV-2. The number of samples and the strength of migration are highly correlated in our analysis. The migration signal may be weak due to insufficient sample. In contrast, the small number of samples could be the result of the low prevalence of DENV-2 and a corresponding weak migratory connection with other regions. To overcome this bias, subsampling is typically conducted to ensure that each region has the same number of sequences.

## Conclusion

Our study provides novel and significant insights into the temporal and spatial dynamics of the DENV-2. The estimated TMRCA suggested that DENV-2 emerged in 1913. Purifying selection is a dominant force acting on E gene to drive the evolution of the virus. Our study suggested that Southeast Asia is the origin of DENV-2 and the hotbed for its spread, and that escalation in genetic diversity lead to epidemics seen in Philippines and Singapore. It is interesting to note that the major routes of viral movement between East&South Asia and Puerto Rico and between South American and Caribbean. The research into virus intercontinental transmission is necessary to understand molecular diversity and to manage public health.

## Materials and Methods

### Sequence dataset

We have downloaded all DENV-2 E gene sequences with known time (year) and geographical locality (country) of isolation from GenBank (http://www.ncbi.nlm.nih.gov/). Strains used in this study are presented in the following format: two letters ISO country code/strain name/year of isolation. In order to minimize the problem of over-representation of some countries, such as the Puerto Rico, 194 sequences were subsampled from the larger set of 946 sequences by location and time to create a more equitable spatiotemporal distribution, excluding sequences with 100% identity and removing the recombinants detected by RDP, Chimaera, BootStan, GENECONV, MaxChi and SiScan methods implemented in RDP4 program^45^ to avoid inferential biases. The sequences, which covers a temporal range of 71 years (1944–2014) from 30 countries, were aligned by MAFFT^46^. The final alignments included 1,485 nucleotides, and were edited manually with Bioedit^47^. The strain names and accession numbers are listed in Table S4. The specific sampling problem is described in detail in the supporting information Text S1.

### Phylogenetic tree inference

Phylogenetic tree was inferred from the alignment using the ML approach implemented in PHYML v3.1^48^ with 100 bootstrap replicates. The best fit substitution model, general-time-reversal incorporating invariant sites and a gamma distribution (GTR + I + G), was chosen by Akaike Information Criterion (AIC) using jModelTest v0.1^49^ (see Text S1 for a detailed comparative analysis). The output from PHYML is mapped onto the reference tree with TreeGradients v1.03^50^, plotting the bootstrap value at the nodes in continuous rainbow color gradient from the root of the tree toward the tips. The existence of temporal information in the data set was tested by regression analysis of root-to-tip genetic distance in the ML (PhyML) tree against sampling date using TempEst v1.5 (http://tree.bio.ed.ac.uk/software/tempest/). The substitution rate remains constant through time, the distance equals the genetic distance from our collected sequences to the root and time equals the sampling time.

### Natural selection

Site-specific selection pressure was assessed using five varying methods implemented in HYPHY 2.2.3^51^ and available through the web-based interface Datamonkey (http://www.datamonkey.org/), including SLAC, FEL, REL, MEME and FUBAR. In these five methods, we utilized TrN model as nucleotide substitution bias model and significance levels set to P < 0.1, Bayes factor >20 or PP > 0.9.

Glycosylation sites were detected using NetNGlyc v1.0^52^ and NetOGlyc v3.1^53^ methods that employ algorithms based on neural networks. Structure homology modeling of E protein was performed using the SWISS-MODEL serve (http://swissmodel.expasy.org). To visualize the locations of glycosylation sites and positive selected sites, each identified amino-acid residue was mapped onto a three-dimensional structure of E protein (Protein Data Bank code: 3J27) using PyMOL (Molecular Graphics System, version 1.7.4 Schrödinger, LLC).

### Time-scaled phylogeography reconstruction

We evaluated the performance of different demographic (constant population size, exponentially growing population and Bayesian skyline plot) and molecular clock (strict and uncorrelated relaxed clock) models by comparing an improved variant of AICM values^54^. To test whether our results are robust to the coalescent prior used, we estimated the TMRCA parameter under six different coalescent prior models. Exponential growth coalescent tree prior is the best model used in the study. The rates of evolutionary (subs/site/year) and TMRCA of DENV-2 were estimated using BEAST v1.8.0 package^55^,^56^, which employs a Bayesian MCMC algorithms to infer population genetic histories from molecular sequences. For this analyses, we used the SRD06 codon position model^57^, to allow partitioning for codon positions (1 + 2 positions and 3 position), with the HKY85 + Γ substitution model applied to these codon divisions. The length of MCMC chain was run for 300 million steps and the log parameter values were sampled at every 30,000 steps after discarding 10% as burn-in. We also used the BEAGLE parallel computation library to enhance the speed of the likelihood calculations^50^. Convergence of parameters was verified with Tracer v.1.5 (available from http://beast.bio.ed.ac.uk/Tracer) by inspecting the Effective Sample Sizes (ESS > 200), and the degree of uncertainty in each parameter estimate is provided by the 95% HPD values. To reconstruct plausible ancestral states, all nodes in the MCC tree were annotated with posterior probability values using TreeAnnotator v.1.8.0, and the tree was visualized with FigTree v1.4.2 (http://tree.bio.ed.ac.uk/software/figtree). The marginal posterior density for the TMRCA is depicted in pink in Fig. 1.

### Estimating population dynamics of Dengue virus type 2

We utilized the BSP, a method for estimating past population dynamics through time from a sample of molecular sequences, without dependence on a prespecified parametric model of demographic history, to assess the change of relative genetic diversity (Neτ; Ne represents the effective population size and τ is generation time) of DENV-2 through time. Then we estimated evolutionary dynamics of DENV-2 isolated from 8 representative geographical regions: Nicaragua, Puerto Rico, Brazil, Caribbean, South America, Southeast Asia, Viet Nam and East&South Asia. To avoid the risk of the model over-parameterization and maximize the reliability of the results, we maintained similar total numbers of sequences for each region across the entire study period. We removed areas with sample size less than 10, and the remaining 188 sequences were used for further analysis. The estimation of the relative change in the population size of DENV-2 over time was made from GMRF Bayesian Skyride coalescent model^58^. For each regional data set, the GMRF skyride plots and the maximum clade credibility trees were reconstructed, and visualized in Tracer and FigTree respectively.

### Discrete phylogeographic analyses and phylogeny-trait association

For understanding the spatial dynamics of DENV-2, phylogeographic analyses were performed to infer diffusion rates and migration patterns simultaneously through time. Three different data sets were used to represent these global epidemic periods: 1964–1990, 1991–2000, and 2001–2013. The asymmetric substitution model with the BSSVS was used to infer asymmetric diffusion rates between any pairwise location state. Meanwhile, BF test was conducted for significance of individual rates to infer the significant epidemiological links. If BF > 3, the phylogeographic link between two locations is considered to be statistically significant and BF > 1,000 is regarded as a very strong support of the degree of rates. The outputs of the Bayesian phylogeographic analyses, as generated by BEAST, were summarized using MCC tree. All demographic parameter settings were the same as in the BEAST analysis described earlier. Further the tree nodes were annotated with their modal location states via color labeling. Then, we used SPREAD v1.06^59^ to convert the estimated divergence times and the spatially-annotated time-scaled phylogeny to a spatiotemporal movement, and visualized the significant migration pathways of the virus.

As the continuous time Markov chain (CTMC) transitions between spatial locations in the phylogeny cannot be directly observed, “Markov jumps” analysis was used to count the number of location state transitions, and the total number of states counts were plotted to present the migration in and out of each location. The waiting times between transitions (Markov rewards) can be tracked on the phylogeny to infer the spatial history of the trunk lineage (described in more detail in Text S1). The program PACT (http://www.trevorbedford.com/pact) was used to perform trunk extraction and processing, which is able to estimate the mean and 95% credible interval for the proportion of the trunk for each geographic region.

To correlate viral phenotypes with phylogeny, we calculated the values of AI, PS and MC based on the posterior samples of trees produced by BEAST v1.8.0 using the Bayesian tip-association significance testing (BaTS)^60^. These statistics methods are described in more detail by Parker *et al*.^60^. A subset of 900 trees was selected from the combined posterior distribution and subsampled using LogCombiner v1.8.0. We used 100 state randomizations to create the null distribution to test the significance of our observed data. In the present study, P value < 0.05 was considered significant for all statistics analyses calculated by BaTS (we have a more detailed description in Text S1).

## Additional Information

**How to cite this article:** Wei, K. and Li, Y. Global evolutionary history and spatio-temporal dynamics of dengue virus type 2. *Sci. Rep.* **7**, 45505; doi: 10.1038/srep45505 (2017).

**Publisher's note:** Springer Nature remains neutral with regard to jurisdictional claims in published maps and institutional affiliations.

## Supplementary Material

Supplemental Information

## Figures and Tables

**Table 1:** Comparison of BEAST models.

Demographic model	Constant			Exponential			BSP		
Clock	Strict	UCED	UCLD	Strict	UCED	UCLD	Strict	UCED	UCLD
**Substitution rate(s/s/y) 95% HPD**	7.31E-4 [6.59E-4, 8.04E-4]	8.17E-4 [6.41E-4, 9.68E-4]	7.78E-4 [6.80E-4, 8.81E-4]	7.29E-4 [6.56E-4, 8.05E-4]	8.91E-4 [7.31E- 4.1.04E-3]	7.90E-4 [6.93E-4, 8.98E-4]	7.35E-4[6.58E-4,8.08E-4]	8.86E-4 [7.36E-4, 1.05E-3]	7.96E-4 [6.96E-4, 9.01E-4]
**TMRCA(root) 95% HPD**	126.66 [110.33, 142.70]	133.79 [74.59.230.30]	123.11 [83.31, 165.87]	124.93 [109.55, 140.85]	101.67 [70.17, 147.78]	112.64 [79.12, 146.88]	124.79 [109.52, 140.65]	103.27 [70.01, 163.60]	110.53 [77.01, 151.22]
**AICM**	24234.39 +/−0.21	24175.410.469	24259.00 +/−0.29	24229.87 +/−0.28	24171.69 +/−0.22	24250.04 +/−0.37	24215.85 +/−0.238	24178.18 +/−0.43	24242.44 +/−0.46

Comparison of parameter estimates and AIC values among combinations of demographic (constant population size, exponential growth and Bayesian skyline-plot) and molecular clock (strict and uncorrelated molecular clock, UCED and UCLD) models for the reconstruction of the evolutionary and demographic history using BEAST.

**Table 2:** Mean and 95% credible intervals over sampled genealogies for the location of the genealogy trunk between the years of 1964 and 2013.

Location	Trunk proportion
**Brazil**	0.066 (0.052, 0.069)
**Caribbean**	0.108 (0.077, 0.138)
**East&South Asia**	0.148 (0.122, 0.179)
**Nicaragua**	0.034 (0.029, 0.041)
**Puerto Rico**	0.126 (0.095, 0.138)
**South America**	0.083 (0.075, 0.094)
**Southeast Asia**	0.294 (0.324, 0.362)
**Viet Nam**	0.110 (0.083, 0.144)

**Table 3:** Phylogeny-trait association tests of the phylogeographic structure of DENV-2 using BaTS.

Statistic	Observed mean	Lower 95% CI	Upper 95% CU	Null mean	Lower 95% CI	Upper 95% CI	Significance
**AI**	9.58	8.60	10.54	16.67	15.32	17.77	0
**PS**	85.80	83	88	127.63	122.20	133.14	0
**MC (state Brazil)**	2	2	2	1.43	1	2	0.26
**MC (state Caribbean)**	3	3	3	2.06	1.31	3	0.09
**MC (state Nicaragua)**	4	4	4	2.01	1.35	3	0.02
**MC (state East&South Asia)**	2	2	2	1.56	1	2	0.33
**MC (state South America)**	2.47	2	4	1.32	1	2	0.16
**MC (state Southeast Asia)**	2.04	1	3	1.37	1	2	0.13
**MC (state Puerto Rico)**	3.02	2	4	1.54	1	2	0.03
**MC (state Viet-Nam)**	2.18	2	3	1.62	1	2.45	0.38
