# Supplementary material for: Global evolutionary history and spatio-temporal dynamics of dengue virus type 2
Source: Sci Rep. 2017 Apr 5;7:45505. doi: 10.1038/srep45505 (PMC5381229; doi:10.1038/srep45505)
Supplement: Supplemental Information [file srep45505-s1.pdf]

Supplementary Information

Global evolutionary history and spatio-temporal dynamics of dengue virus type 2

Kaifa Wei, Yuhan Li

School of Biological Sciences and Biotechnology, Minnan Normal University, Zhangzhou 363000, China

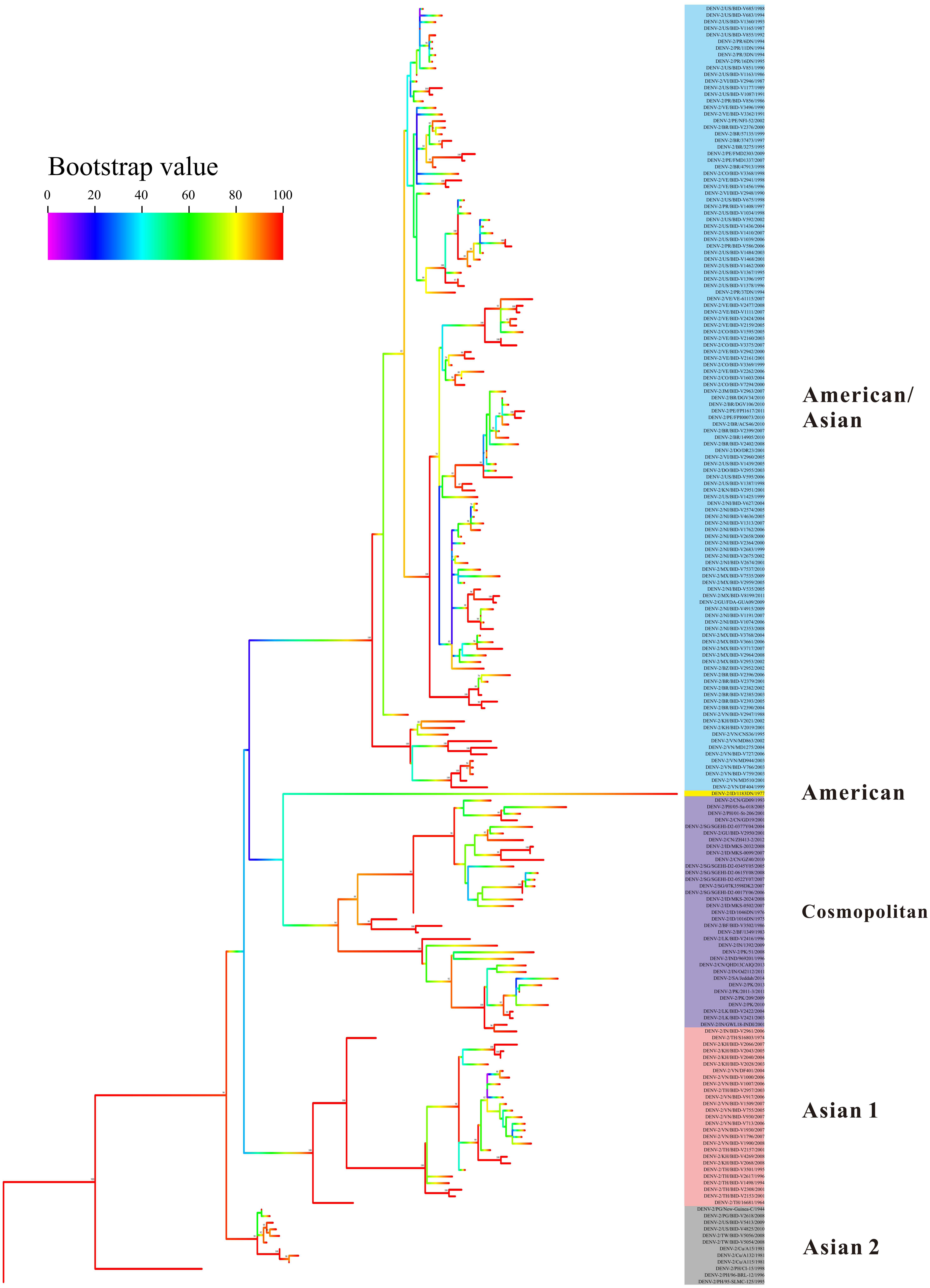

Figure S1. Maximum likelihood phylogeny of DENV-2 E gene sequences from 1944 to 2014 and the color gradient for the branches represents bootstrap values. The five subtypes are indicated on the right side of the tree.

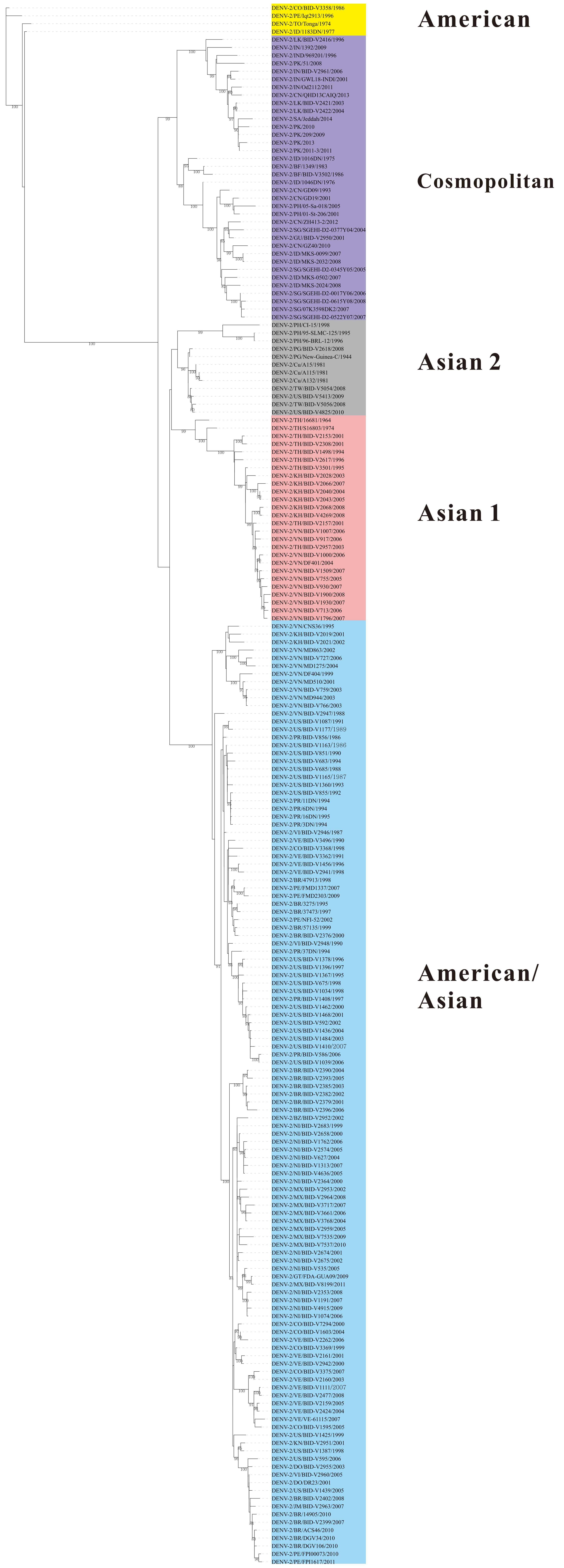

Figure S2. Maximum likelihood phylogeny including 194 isolates of DENV-2 E gene plus 3 American genotype reference sequences. The five subtypes are indicated on the right side of the tree.

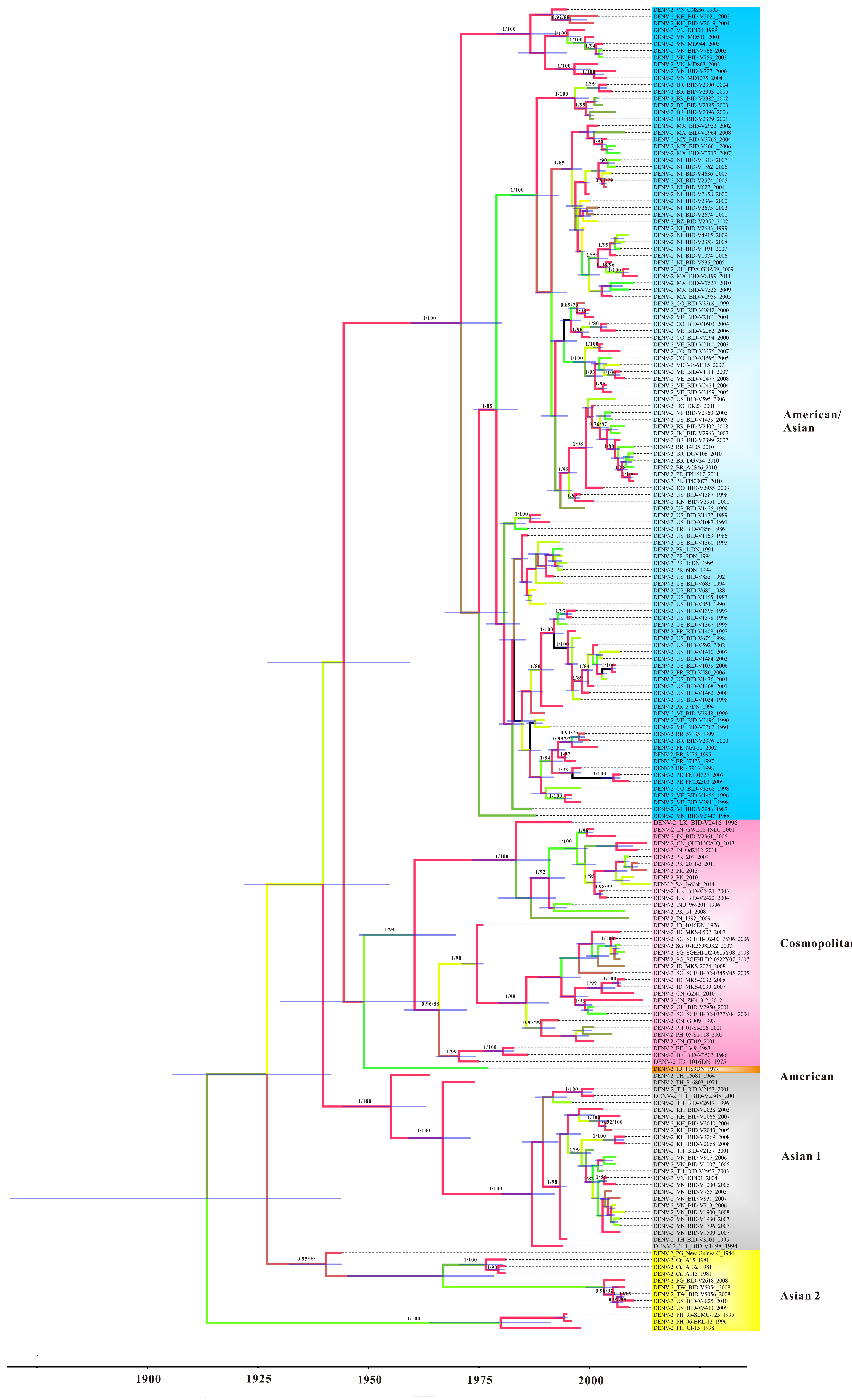

Figure S3. Maximum clade credibility (MCC) tree of Dengue virus type 2. Maximum clade credibility tree derived from the Bayesian analysis of the envelope protein of DENV-2 with the best fit model (relaxed exponential clock). DENV-2 genotypes (American, Asian 1 and 2, Cosmopolitan and American/Asian) are identified. The bootstrap (BS) values and posterior probability (PP) values for the key nodes are indicated as BS/PP.

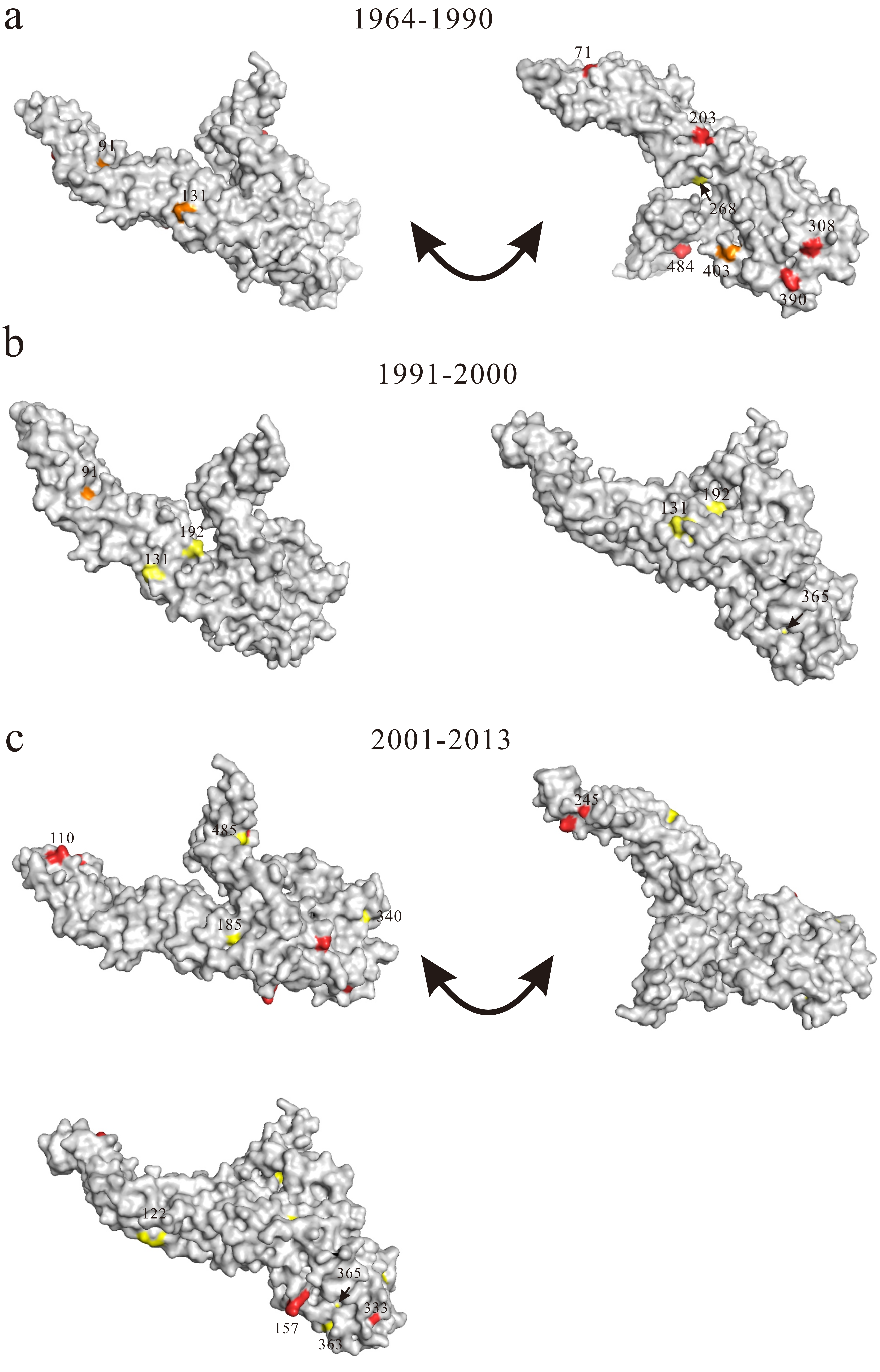

Figure S4. Mapping of positively selected amino-acid sites onto the three-dimensional structure of the E protein of DENV-2 during 1964-1990, 1991-2000 and 2001-2013, respectively. Yellow indicates positive selection site detected by the MEME method; The red color indicates positive selection site detected by the REL method; The orange color indicates positive selection sites that are detected by both methods.

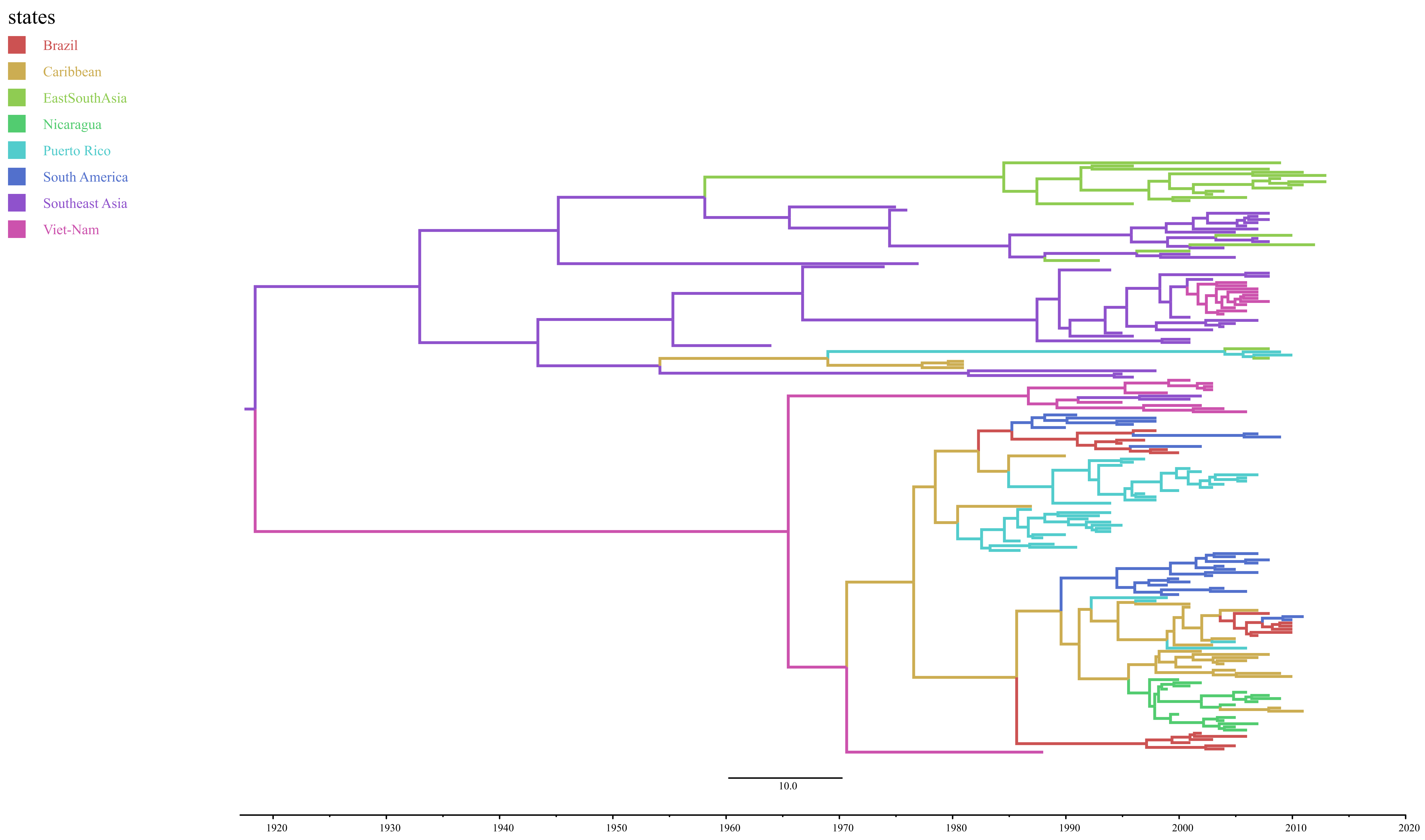

Figure S5. Bayesian phylogeographic tree of DENV-2 E gene sequences. Geographic locations showed with different colors in the tree are represent in the legend on the left.

a

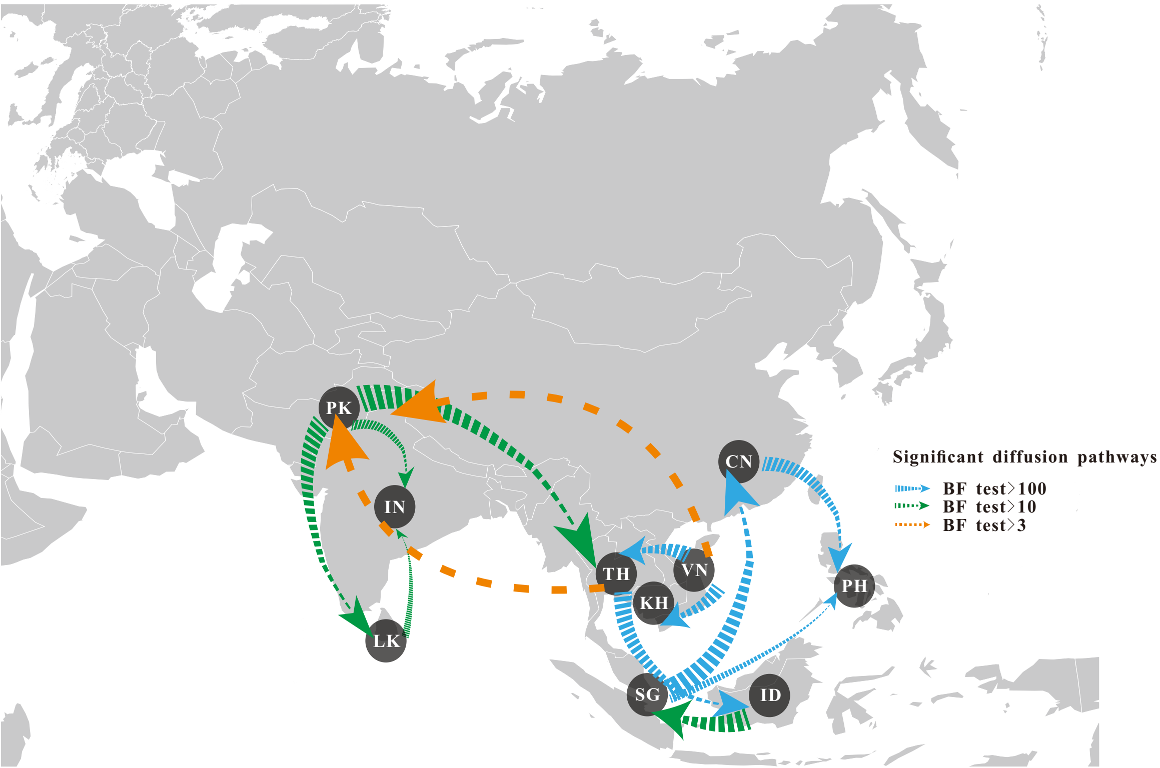

b

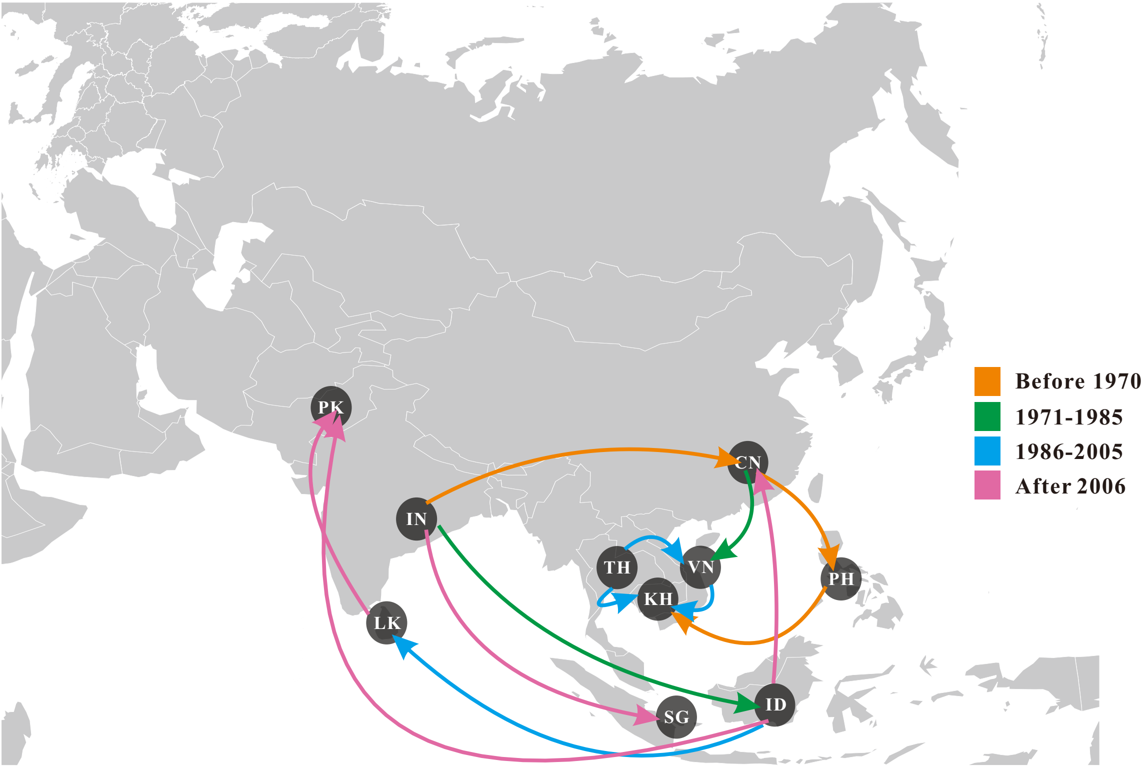

Figure S6. Spatiotemporal dynamics of dissemination of DENV-2. **a.** Significant pathways from one location to another are indicated on the maps. Blue arrow represents strongly supported rate with 100≤BF<1000; Dark green arrow, supported rate with 3≤BF<100; Orange arrow represents the support with BF>3. PK, Pakistan; IN, India; LK, Sri Lanka; CN, China; PH, Philippines; TH, Thailand; VN, Viet Nam; KH, Cambodia; SG, Singapore; ID, Indonesia. **b.** Viral dispersal pattern between 1964 and 2013. Lines between locations represent branches in the Bayesian MCC tree along which location transitions occurs. The color of lines informs the date of the viral migrations among each pair of locations. The map source is downloaded freely from FREE WORLD MAPS (http://www.freeworldmaps.net/pdf/asia/asia_countries.pdf).

Table S1. Selection pressures of different periods for E gene of globally sampled DENV-2 virus.

Time	SLAC	FEL	REL	MEME	FUBAR
1964-1990	-	-	71,91,131,203,308, 390,403,484	91,131,268,403	-
1991-2000	-	91	-	91,131,192,365	-
2001-2013	-	-	110,157,245,333	122,185,340,363,365,485	-

Table S2. A list of predicted the N-linked and O-linked glycosylation sites in E protein of DENV-2.

	Glycosylation site
N-linked	67 (NTTT), 153 (NDTG)
O-linked	S (32, 33, 40, 66, 69, 70, 76, 138, 142, 165, 236, 265, 353, 359, 405)
	T (168, 169, 186, 274, 363, 397, 431)

Table S3. Significant viral migration pathways between pairs of geographic locations. Links supported by Bayes Factor (BF) values.

**Transition Between**		**Mean actual rate**	**Mean indicator**	**Bayes Factor**
**From**	**To**			
**South America**	**Caribbean**	0.926	0.123	>1000
**Southeast Asia**	**Viet-Nam**	0.953	0.095	3-100
**Southeast Asia**	**Brazil**	0.939	0.066	3-100
**Southeast Asia**	**Caribbean**	0.965	0.065	3-100
**Puerto Rico**	**Brazil**	0.977	0.065	100-1000
**East&South Asia**	**Nicaragua**	0.985	0.057	100-1000
**Southeast Asia**	**South America**	1.964	1	3-100
**Caribbean**	**South America**	0.969	0.164	100-1000
**Viet-Nam**	**Southeast Asia**	0.97	0.071	3-100
**East&South Asia**	**Southeast Asia**	1.302	0.944	3-100
**Nicaragua**	**Southeast Asia**	0.983	0.032	3-100
**Brazil**	**Puerto Rico**	0.962	0.092	3-100
**East&South Asia**	**Puerto Rico**	0.991	0.076	>1000
**Nicaragua**	**East&South Asia**	0.872	0.34	100-1000

Table S4. Accession numbers of the sequences used in this study.

Accession	Strain name	Years	Country	Host
**KF744397**	DENV-2/PH/01-St-206/2001	2001	Philippines	Human
**KF744398**	DENV-2/PH/05-Sa-018/2005	2005	Philippines	Human
**EU056810**	DENV-2/BF/1349/1983	1983	Burkina-Faso	Human
**JX475906**	DENV-2/IN/1392/2009	2009	India	Human
**JX669477**	DENV-2/BR/14905/2010	2010	Brazil	Human
**NC-001474**	DENV-2/TH/16681/1964	1964	Thailand	Unknown
**JX669480**	DENV-2/BR/3275/1995	1995	Brazil	Human
**JX669483**	DENV-2/BR/37473/1997	1997	Brazil	Human
**JX669484**	DENV-2/BR/47913/1998	1998	Brazil	Human
**JX669486**	DENV-2/BR/57135/1999	1999	Brazil	Human
**KF744403**	DENV-2/PH/95-SLMC-125/1995	1995	Philippines	Human
**KF744404**	DENV-2/PH/96-BRL-12/1996	1996	Philippines	Human
**KF744399**	DENV-2/PH/CI-15/1998	1998	Philippines	Human
**JX286516**	DENV-2/BR/ACS46/2010	2010/3/1	Brazil	Human
**JX649148**	DENV-2/VN/CNS36/1995	1995	Viet-Nam	Human
**KF704356**	DENV-2/Cu/A115/1981	1981	Cuba	Human
**KF704355**	DENV-2/Cu/A132/1981	1981	Cuba	Human
**KF704354**	DENV-2/Cu/A15/1981	1981	Cuba	Human
**KF041233**	DENV-2/PK/2011-3/2011	2011	Pakistan	Human
**KF041235**	DENV-2/PK/209/2009	2009	Pakistan	Human
**KF041236**	DENV-2/PK/51/2008	2008	Pakistan	Human
**GU131843**	DENV-2/BF/BID-V3502/1986	1986	Burkina-Faso	Mosquito
**FJ850072**	DENV-2/BR/BID-V2376/2000	2000	Brazil	Human
**FJ850074**	DENV-2/BR/BID-V2379/2001	2001	Brazil	Human
**FJ850076**	DENV-2/BR/BID-V2382/2002	2002	Brazil	Human
**GQ868640**	DENV-2/BR/BID-V2385/2003	2003	Brazil	Human
**FJ850082**	DENV-2/BR/BID-V2390/2004	2004	Brazil	Human
**FJ850085**	DENV-2/BR/BID-V2393/2005	2005	Brazil	Human
**FJ850088**	DENV-2/BR/BID-V2396/2006	2006	Brazil	Human
**FJ850091**	DENV-2/BR/BID-V2399/2007	2007	Brazil	Human
**GQ199890**	DENV-2/BR/BID-V2402/2008	2008	Brazil	Human
**FJ898461**	DENV-2/BZ/BID-V2952/2002	2002	Belize	Human
**FJ024474**	DENV-2/CO/BID-V1595/2005	2005	Colombia	Human
**FJ024477**	DENV-2/CO/BID-V1603/2004	2004	Colombia	Human
**GQ868552**	DENV-2/CO/BID-V3368/1998	1998	Colombia	Human
**GQ868553**	DENV-2/CO/BID-V3369/1999	1999	Colombia	Human
**GQ868558**	DENV-2/CO/BID-V3375/2007	2007	Colombia	Human
**KJ189305**	DENV-2/CO/BID-V7294/2000	2000	Colombia	Human
**FJ898451**	DENV-2/DO/BID-V2955/2003	2003	Dominican-Republic	Human
**HM488257**	DENV-2/GU/BID-V2950/2001	2001	Guam	Human
**HQ999999**	DENV-2/GU/FDA-GUA09/2009	2009	Guatemala	Human
**GQ398258**	DENV-2/ID/1016DN/1975	1975	Indonesia	Human

**GQ398264**	DENV-2/ID/1046DN/1976	1976	Indonesia	Human
**GQ398257**	DENV-2/ID/1183DN/1977	1977	Indonesia	Human
**FJ898454**	DENV-2/IN/BID-V2961/2006	2006	India	Human
**JQ922549**	DENV-2/IN/969201/1996	1996	India	Human
**GQ199892**	DENV-2/JM/BID-V2963/2007	2007	Jamaica	Human
**JF730044**	DENV-2/KH/BID-V2019/2001	2001	Cambodia	Human
**FJ639698**	DENV-2/KH/BID-V2021/2002	2002	Cambodia	Human
**GQ868620**	DENV-2/KH/BID-V2028/2003	2003	Cambodia	Human
**FJ639706**	DENV-2/KH/BID-V2040/2004	2004	Cambodia	Human
**FJ639709**	DENV-2/KH/BID-V2043/2005	2005	Cambodia	Human
**FJ639717**	DENV-2/KH/BID-V2066/2007	2007	Cambodia	Human
**FJ639718**	DENV-2/KH/BID-V2068/2008	2008	Cambodia	Human
**KF955401**	DENV-2/KH/BID-V4269/2008	2008	Cambodia	Human
**FJ898460**	DENV-2/KN/BID-V2951/2001	2001	Saint-Kitts-and-Nevis	Human
**FJ882602**	DENV-2/LK/BID-V2416/1996	1996	Sri-Lanka	Human
**GQ252676**	DENV-2/LK/BID-V2421/2003	2003	Sri-Lanka	Human
**GQ252677**	DENV-2/LK/BID-V2422/2004	2004	Sri-Lanka	Human
**FJ898438**	DENV-2/MX/BID-V2953/2002	2002	Mexico	Human
**GQ199894**	DENV-2/MX/BID-V2959/2005	2005	Mexico	Human
**FJ898439**	DENV-2/MX/BID-V2964/2008	2008	Mexico	Human
**GU131959**	DENV-2/MX/BID-V3661/2006	2006	Mexico	Human
**GU131975**	DENV-2/MX/BID-V3717/2007	2007	Mexico	Human
**GU131955**	DENV-2/MX/BID-V3768/2004	2004	Mexico	Human
**KJ189308**	DENV-2/MX/BID-V7535/2009	2009	Mexico	Mosquito
**KJ189309**	DENV-2/MX/BID-V7537/2010	2010	Mexico	Mosquito
**KJ189370**	DENV-2/MX/BID-V8199/2011	2011	Mexico	Human
**EU482620**	DENV-2/NI/BID-V1074/2006	2006	Nicaragua	Human
**EU482621**	DENV-2/NI/BID-V1191/2007	2007	Nicaragua	Human
**FJ882594**	DENV-2/NI/BID-V1313/2007	2007	Nicaragua	Human
**FJ744741**	DENV-2/NI/BID-V1762/2006	2006	Nicaragua	Human
**FJ810418**	DENV-2/NI/BID-V2353/2008	2008	Nicaragua	Human
**FJ744744**	DENV-2/NI/BID-V2364/2000	2000	Nicaragua	Human
**FJ850053**	DENV-2/NI/BID-V2574/2005	2005	Nicaragua	Human
**FJ850118**	DENV-2/NI/BID-V2658/2000	2000	Nicaragua	Human
**FJ850121**	DENV-2/NI/BID-V2674/2001	2001	Nicaragua	Human
**GQ199897**	DENV-2/NI/BID-V2675/2002	2002	Nicaragua	Human
**GQ199895**	DENV-2/NI/BID-V2683/1999	1999	Nicaragua	Human
**HQ541793**	DENV-2/NI/BID-V4636/2005	2005	Nicaragua	Human
**HQ705625**	DENV-2/NI/BID-V4915/2009	2009	Nicaragua	Human
**EU482757**	DENV-2/NI/BID-V535/2005	2005	Nicaragua	Human
**FJ898436**	DENV-2/NI/BID-V627/2004	2004	Nicaragua	Human
**KC294200**	DENV-2/PE/FMD1337/2007	2007/3/28	Peru	Human
**KC294201**	DENV-2/PE/FMD2303/2009	2009/2/13	Peru	Human
**KC294202**	DENV-2/PE/FPI00073/2010	2010/12/13	Peru	Human

**KC294210**	DENV-2/PE/FPI1617/2011	2011/3/23	Peru	Human
**KC294222**	DENV-2/PE/NFI-52/2002	2002/3/22	Peru	Human
**FJ906959**	DENV-2/PG/BID-V2618/2008	2008	Papua-New-Guinea	Human
**KF360005**	DENV-2/PK/2010	2010/11/1	Pakistan	Human
**KJ010186**	DENV-2/PK/2013	2013/10/2	Pakistan	Human
**GQ398313**	DENV-2/PR/11DN/1994	1994	Puerto-Rico	Human
**GQ398298**	DENV-2/PR/16DN/1995	1995	Puerto-Rico	Human
**GQ398283**	DENV-2/PR/37DN/1994	1994	Puerto-Rico	Human
**GQ398308**	DENV-2/PR/3DN/1994	1994	Puerto-Rico	Human
**GQ398292**	DENV-2/PR/6DN/1994	1994	Puerto-Rico	Human
**KF955373**	DENV-2/PR/BID-V1408/1997	1997	Puerto-Rico	Human
**KF955359**	DENV-2/PR/BID-V586/2006	2006	Puerto-Rico	Human
**KF955363**	DENV-2/PR/BID-V856/1986	1986	Puerto-Rico	Human
**GQ398266**	DENV-2/SG/07K3598DK2/2007	2007	Singapore	Human
**EU687246**	DENV-2/TH/BID-V1498/1994	1994	Thailand	Human
**FJ639828**	DENV-2/TH/BID-V2153/2001	2001	Thailand	Human
**FJ639832**	DENV-2/TH/BID-V2157/2001	2001	Thailand	Human
**FJ744722**	DENV-2/TH/BID-V2308/2001	2001	Thailand	Human
**FJ906958**	DENV-2/TH/BID-V2617/1996	1996	Thailand	Human
**FJ898452**	DENV-2/TH/BID-V2957/2003	2003	Thailand	Human
**GQ868543**	DENV-2/TH/BID-V3501/1995	1995	Thailand	Human
**HQ891023**	DENV-2/TW/BID-V5054/2008	2008	Taiwan	Human
**HQ891024**	DENV-2/TW/BID-V5056/2008	2008	Taiwan	Mouse
**EU482547**	DENV-2/US/BID-V1034/1998	1998	USA	Human
**EU482551**	DENV-2/US/BID-V1039/2006	2006	USA	Human
**EU529701**	DENV-2/US/BID-V1087/1991	1991	USA	Human
**EU569704**	DENV-2/US/BID-V1163/1986	1986	USA	Human
**EU482569**	DENV-2/US/BID-V1165/1987	1987	USA	Human
**EU482580**	DENV-2/US/BID-V1177/1989	1989	USA	Human
**EU482589**	DENV-2/US/BID-V1360/1993	1993	USA	Human
**EU569706**	DENV-2/US/BID-V1367/1995	1995	USA	Human
**EU596485**	DENV-2/US/BID-V1378/1996	1996	USA	Human
**EU687212**	DENV-2/US/BID-V1387/1998	1998	USA	Human
**EU569714**	DENV-2/US/BID-V1396/1997	1997	USA	Human
**EU596488**	DENV-2/US/BID-V1410/2007	2007	USA	Human
**EU677142**	DENV-2/US/BID-V1425/1999	1999	USA	Human
**EU687215**	DENV-2/US/BID-V1436/2004	2004	USA	Human
**EU687216**	DENV-2/US/BID-V1439/2005	2005	USA	Human
**EU687223**	DENV-2/US/BID-V1462/2000	2000	USA	Human
**EU687228**	DENV-2/US/BID-V1468/2001	2001	USA	Human
**EU687236**	DENV-2/US/BID-V1484/2003	2003	USA	Human
**HQ541799**	DENV-2/US/BID-V4825/2010	2010	USA	Mouse
**JF730054**	DENV-2/US/BID-V5413/2009	2009	USA	Mouse
**EU482723**	DENV-2/US/BID-V592/2002	2002	USA	Human

**EU482726**	DENV-2/US/BID-V595/2006	2006	USA	Human
**EU482732**	DENV-2/US/BID-V675/1998	1998	USA	Human
**EU482740**	DENV-2/US/BID-V683/1994	1994	USA	Human
**EU482742**	DENV-2/US/BID-V685/1988	1988	USA	Human
**EU482590**	DENV-2/US/BID-V851/1990	1990	USA	Human
**EU482594**	DENV-2/US/BID-V855/1992	1992	USA	Human
**EU482608**	DENV-2/VE/BID-V1111/2007	2007	Venezuela	Human
**EU687220**	DENV-2/VE/BID-V1456/1996	1996	Venezuela	Human
**FJ639733**	DENV-2/VE/BID-V2159/2005	2005	Venezuela	Human
**FJ639734**	DENV-2/VE/BID-V2160/2003	2003	Venezuela	Human
**JN819408**	DENV-2/VE/BID-V2161/2001	2001	Venezuela	Human
**FJ639822**	DENV-2/VE/BID-V2262/2006	2006	Venezuela	Human
**FJ850112**	DENV-2/VE/BID-V2424/2004	2004	Venezuela	Human
**FJ850107**	DENV-2/VE/BID-V2477/2008	2008	Venezuela	Human
**FJ898465**	DENV-2/VE/BID-V2941/1998	1998	Venezuela	Human
**FJ898466**	DENV-2/VE/BID-V2942/2000	2000	Venezuela	Human
**GQ868595**	DENV-2/VE/BID-V3362/1991	1991	Venezuela	Human
**GQ868540**	DENV-2/VE/BID-V3496/1990	1990	Venezuela	Human
**GQ868603**	DENV-2/VI/BID-V2946/1987	1987	Virgin-Islands	Human
**FJ898450**	DENV-2/VI/BID-V2948/1990	1990	Virgin-Islands	Human
**FJ898453**	DENV-2/VI/BID-V2960/2005	2005	Virgin-Islands	Human
**EU482445**	DENV-2/VN/BID-V1000/2006	2006	Viet-Nam	Human
**EU482451**	DENV-2/VN/BID-V1007/2006	2006	Viet-Nam	Human
**EU687248**	DENV-2/VN/BID-V1509/2007	2007	Viet-Nam	Human
**FJ859028**	DENV-2/VN/BID-V1796/2007	2007	Viet-Nam	Human
**FJ410237**	DENV-2/VN/BID-V1900/2008	2008	Viet-Nam	Human
**FJ547067**	DENV-2/VN/BID-V1930/2007	2007	Viet-Nam	Human
**JN819418**	DENV-2/VN/BID-V2947/1988	1988	Viet-Nam	Human
**EU482650**	DENV-2/VN/BID-V713/2006	2006	Viet-Nam	Human
**EU482664**	DENV-2/VN/BID-V727/2006	2006	Viet-Nam	Human
**EU482777**	DENV-2/VN/BID-V755/2005	2005	Viet-Nam	Human
**EU482781**	DENV-2/VN/BID-V759/2003	2003	Viet-Nam	Human
**EU482788**	DENV-2/VN/BID-V766/2003	2003	Viet-Nam	Human
**EU482463**	DENV-2/VN/BID-V917/2006	2006	Viet-Nam	Human
**EU482475**	DENV-2/VN/BID-V930/2007	2007	Viet-Nam	Human
**FM210216**	DENV-2/VN/DF401/2004	2004	Viet-Nam	Human
**FM210217**	DENV-2/VN/DF404/1999	1999	Viet-Nam	Human
**JX286524**	DENV-2/BR/DGV106/2010	2010/4/15	Brazil	Human
**JX286522**	DENV-2/BR/DGV34/2010	2010/2/24	Brazil	Human
**AB122020**	DENV-2/DO/DR23/2001	2001	Dominican-Republic	Human
**KC964094**	DENV-2/CN/GD09/1993	1993	China	Human
**KC964093**	DENV-2/CN/GD19/2001	2001	China	Human
**DQ448231**	DENV-2/IN/GWL18-INDI/2001	2001	India	Human
**JX470186**	DENV-2/CN/GZ40/2010	2010	China	Human

**KJ830750**	DENV-2/SA/Jeddah/2014	2014/1/13	Saudi-Arabia	Human
**FM210241**	DENV-2/VN/MD1275/2004	2004	Viet-Nam	Human
**FM210238**	DENV-2/VN/MD510/2001	2001	Viet-Nam	Human
**FM210227**	DENV-2/VN/MD863/2002	2002	Viet-Nam	Human
**FM210230**	DENV-2/VN/MD944/2003	2003	Viet-Nam	Human
**KC762676**	DENV-2/ID/MKS-0099/2007	2007/7/17	Indonesia	Human
**KC762656**	DENV-2/ID/MKS-0502/2007	2007/12/11	Indonesia	Human
**KC762663**	DENV-2/ID/MKS-2024/2008	2008/2/20	Indonesia	Human
**KC762675**	DENV-2/ID/MKS-2032/2008	2008/3/8	Indonesia	Human
**KM204118**	DENV-2/PG/New-Guinea-C/1944	1944	Papua-New-Guinea	Human
**JQ955624**	DENV-2/IN/Od2112/2011	2011	India	Human
**KF479233**	DENV-2/CN/QHD13CAIQ/2013	2013/1/4	China	Human
**GU289914**	DENV-2/TH/S16803/1974	1974	Thailand	Human
**JN851113**	DENV-2/SG/SGEHI-D2-0017Y06/2006	2006	Singapore	Human
**JN851125**	DENV-2/SG/SGEHI-D2-0345Y05/2005	2005	Singapore	Human
**JN851123**	DENV-2/SG/SGEHI-D2-0377Y04/2004	2004	Singapore	Human
**JN851114**	DENV-2/SG/SGEHI-D2-0522Y07/2007	2007	Singapore	Human
**JN851121**	DENV-2/SG/SGEHI-D2-0615Y08/2008	2008	Singapore	Human
**HQ332186**	DENV-2/VE/VE-61115/2007	2007	Venezuela	Human
**KC131142**	DENV-2/CN/ZH413-2/2012	2012/9/1	China	Human

Text S1

Supplementary Methods:

Sequence dataset

E gene sequences for Dengue virus type 2 collected by the National Center for Biotechnology Information (NCBI) (http://www.ncbi.nlm.nih.gov/) between 1944 and 2014. After removing duplicate strains and recombination sequences, the data set contained 946 DENV-2 sequences. Sampling locations for these sequences were parsed from strain names. Some regions in the original sequence data, such as Puerto Rico, are over-represented. In addition, the strains in recent years have a higher sample size compared to the beginning of the study period. To control these sampling biases, we subsampled the raw data randomly based on location and time to create a more equitable spatiotemporal distribution. Selected sequences to ensure the maximization of the available sequences within each region while still providing sufficient geographical diversity to ensure almost global coverage. From 1986 to 2010, Puerto Rico had many available sequences each year, so it was necessary to sample fewer sequences per year from the Puerto Rico in order to maintain a similar total number of sequences for each region throughout the study. We selected nor more than 5 sequences per region per year for the virus. When selecting a subsampled sequence, we first selected the sequences with the complete year and then the longer sequences to exclude the incomplete sequences. Finally, E gene sequence data for 194 DENV-2 was obtained and used for the next analysis.

Phylogenetic Inference

Maximum likelihood (ML) phylogenetic trees were constructed in PhyML v 3.1[1] with 100 bootstrap replicates. A general time-reversible model of nucleotide substitution was implemented, with gamma distributed rate heterogeneity of 4 rate categories and a proportion of invariable sites. Then, the phylogeny of DENV-2 was generated using the Bayesian Monte Carlo Markov Chain (MCMC) method implemented in BEAST v.1.8.0[2]. Different combinations of substitution models, clock models, and population size models were compared using a posterior simulation-based analogue of Akaike's information reiteration through MCMC (AICM), the AIC was measured from a posterior of each model in a Bayesian Monte Carlo context[3]. The comparisons were conducted in Tracer v1.5 (http://beast.bio.ed.ac.uk) using a log file of each model. For our dataset, the best-fit model incorporated a SRD06[4] nucleotide substitution model with uncorrelated exponential relaxed

molecular clocks and an exponential growth population coalescent process prior over the phylogeny. This combined model is also used to estimate subsequent evolutionary rates and divergent times. MCMC chains were run for 300 million steps sampled every 10,000 states with 10% burn-in. MCMC convergence and effective sample size of parameter estimates were evaluated using Tracer 1.5. Maximum clade credibility (MCC) tree were summarized using Tree Annotator and visualized using FigTree v1.4.2 (http://tree.bio.ed.ac.uk/software/figtree/). The overall rates of evolutionary change (nucleotide substitutions per site per year (subs/site/year)) and tree root ages were estimated simultaneously.

Discrete phylogeographic analyses

The phylogeographic analysis considered 8 locations: Nicaragua, Puerto Rico, Brazil, Caribbean, South America, Southeast Asia, Viet Nam and East&South Asia. To determine the viral dissemination network among geographical regions, an asymmetric discrete phylogeography model was applied. We employ a continuous time Markov chain (CTMC)[5] phylogeographic model implemented in BEAST, coupled with model averaging using a Bayesian stochastic search variable selection (BSSVS) procedure[5]. BSSVS is a measure of statistical support for the rate of discrete traits and that enables us to compute a Bayes factor (BF) test for identifying the most parsimonious diffusion routes. In order to reduce the risk of model over-parameterization, the simplest model that the HKY nucleotide substitution model with strict clock and a constant-population coalescent process can be used. This tree was treated as independent draws from the posterior space of trees when subsequently used in the phylogeographic analyses. The resulting log files were used to calculate the BF for the diffusion between discrete locations, and to extract the actual non-zero rates and mean indicators for all statistically supported routes. Significant migration routes were summarized based on BF>3. We defined the degree of rate support as follows: BF>1,000 indicates decisive, 100<BF<1,000 indicates very strong support, 10<BF<100 indicates strong support and 3< BF<10 indicates supported.

Recent advances in explicit calculation[6] enable computation of the expected number of transitions (jumps) and waiting times in given locations (rewards). 'Markov jump' counts[7] of the expected number of location state transitions along the phylogenetic branches provide a quantitative measure of gene flow between regions, representing successful viral introductions from one region to another. To quantify the dissemination process, we estimated the rate of DENV-2 transmission in the sampled

isolates using the 'Markov jump' counts of location state transitions for all possible states along the phylogeny. Then we used the time between state changes (Markov reward) to estimate the duration the ancestral population occupies a particular location. That is to say, the jumps quantify the transitions, whereas the rewards quantify the time spent in any particular state (on the phylogeny).

Phylogeny-trait association

We quantify spatial signal using Bayesian tip-association tests, implemented through the Bayesian tip-association significance testing (BaTS) software package. To determine the overall degree of geographical structure among the DENV-2 viruses, we assign each sequence to the eight geographical regions and compute association index (AI)[8] and parsimony score (PS)[9] using BaTS on a subset of 100 samples from the posterior distribution of topologies. The first two statistics are to assess the overall extent of clustering for the mutations of interest in the phylogenetic tree. Additionally, the monophyletic clade (MC)[10] statistic allows the user to test for an association between individual geographical regions and viruses. The observed mean and its associated 95% confidence intervals (Upper and Lower CI) were obtained by analyzing trees sampled during the Bayesian phylogenetic reconstruction. In the present study, P values < 0.05 were considered significant for all statistics calculated by BaTS.

Reference:

1 Guindon, S. & Gascuel, O. A simple, fast, and accurate algorithm to estimate large phylogenies by maximum likelihood. *Systematic biology* **52**, 696-704 (2003).

2 Drummond, A. J., Suchard, M. A., Xie, D. & Rambaut, A. Bayesian phylogenetics with BEAUti and the BEAST 1.7. *Molecular biology and evolution* **29**, 1969-1973 (2012).

3 Baele, G. *et al.* Improving the accuracy of demographic and molecular clock model comparison while accommodating phylogenetic uncertainty. *Molecular biology and evolution* **29**, 2157-2167 (2012).

4 Shapiro, B., Rambaut, A. & Drummond, A. J. Choosing appropriate substitution models for the phylogenetic analysis of protein-coding sequences. *Molecular biology and evolution* **23**, 7-9 (2006).

5 Lemey, P., Rambaut, A., Drummond, A. J. & Suchard, M. A. Bayesian phylogeography finds its roots. *PLoS computational biology* **5**, e1000520 (2009).

6 Minin, V. N. & Suchard, M. A. Fast, accurate and simulation-free stochastic mapping. *Philosophical transactions of the Royal Society of London. Series B, Biological sciences* **363**, 3985-3995 (2008).

7 Minin, V. N. & Suchard, M. A. Counting labeled transitions in continuous-time Markov models of evolution. *Journal of mathematical biology* **56**, 391-412 (2008).

8 Wang, T. H., Donaldson, Y. K., Brettle, R. P., Bell, J. E. & Simmonds, P. Identification of shared populations of human immunodeficiency virus type 1 infecting microglia and tissue macrophages outside the central nervous system. *J Virol* **75**, 11686-11699 (2001).

9 Slatkin, M. & Maddison, W. P. A cladistic measure of gene flow inferred from the phylogenies of alleles. *Genetics* **123**, 603-613 (1989).

10 Parker, J., Rambaut, A. & Pybus, O. G. Correlating viral phenotypes with phylogeny: accounting for phylogenetic uncertainty. *Infection, Genetics and Evolution* **8**, 239-246 (2008).
